# Synthesis, Characterization, and Reactivity of a Uranium(VI) Carbene Imido Oxo Complex**

**DOI:** 10.1002/anie.201403892

**Published:** 2014-05-19

**Authors:** Erli Lu, Oliver J Cooper, Jonathan McMaster, Floriana Tuna, Eric J L McInnes, William Lewis, Alexander J Blake, Stephen T Liddle

**Affiliations:** School of Chemistry, University of Nottingham, University ParkNottingham, NG7 2RD (UK); School of Chemistry and Photon Science Institute, University of ManchesterOxford Road, Manchester, M13 9PL (UK)

**Keywords:** carbene ligands, imido ligands, multiple bonding, oxo ligands, uranium

## Abstract

We report the uranium(VI) carbene imido oxo complex [U(BIPM^TMS^)(NMes)(O)(DMAP)_2_] (**5**, BIPM^TMS^=C(PPh_2_NSiMe_3_)_2_; Mes=2,4,6-Me_3_C_6_H_2_; DMAP=4-(dimethylamino)pyridine) which exhibits the unprecedented arrangement of three formal multiply bonded ligands to one metal center where the coordinated heteroatoms derive from different element groups. This complex was prepared by incorporation of carbene, imido, and then oxo groups at the uranium center by salt elimination, protonolysis, and two-electron oxidation, respectively. The oxo and imido groups adopt axial positions in a T-shaped motif with respect to the carbene, which is consistent with an inverse *trans*-influence. Complex **5** reacts with *tert*-butylisocyanate at the imido rather than carbene group to afford the uranyl(VI) carbene complex [U(BIPM^TMS^)(O)_2_(DMAP)_2_] (**6**).

There is burgeoning interest in covalent uranium–ligand (UL) multiple bonding because of the ongoing debate regarding the level and nature of covalency that these bonds may exhibit.[1] Uranium UL compounds containing covalent terminal monocarbene, -imido, -nitride, and -chalcogenide linkages are well known.[2–5] Homoleptic UL_2_ compounds are also well represented; in addition to a modest range of uranium biscarbene and bisimido complexes,[2b,d, 6] the bisoxo uranyl unit accounts for more than 50 % of all structurally characterized uranium complexes.[7] Recently, progress has been made preparing heteroleptic ULL′ complexes with carbene–oxo,[2k] imido–oxo,[8] nitrido–oxo,[9] and heavier chalcogen–oxo compounds.[10] Although both rare and challenging to prepare, these compounds are of interest with respect to the inverse *trans*-influence (ITI),[11] where strong donor ligands adopt *trans* geometries in contrast to d-block analogues that tend to adopt *cis* geometries. Concerning three-ligand multiple-bond linkages to uranium, examples are limited to homoleptic systems, such as uranium trioxide and triscarbenes,[2c] or heteroleptic UL_2_L′ systems with a maximum of two different types of multiply bonded ligands, such as a uranyl–carbene.[2f] Remarkably, no heteroleptic ULL′L′′ uranium complexes containing three different multiple bond linkages to uranium have ever been reported. Furthermore, even for d-block complexes where metal–ligand (ML) multiple bonding is more favorable, homoleptic combinations are so dominant that there are no examples of MLL′L′′ multiply bonded complexes containing heteroatoms from different element groups; the only example of a MLL′L′′ complex from hundreds of examples of ML multiple bond complexes is the all-chalcogen complex [W(C_5_Me_5_)(O)(S)(Se)][PPh_4_], reported over a decade ago.[12] This paucity may reflect the difficulties of constructing different covalent ML multiple bonds at a metal center whilst avoiding decomposition of previously installed multiple bonds. Although MLL′L′′ complexes utilizing heteroatoms from different element groups are yet to be reported, their synthesis would establish new synthetic strategies, give structure–bonding insights, and allow competitive reactivity studies to be investigated.

Herein, we describe the synthesis of a uranium(VI) carbene imido oxo complex, which is the first example of a metal complex to exhibit formal covalent multiple-bond interactions to three different ligands with heteroatoms from different element groups, and we describe its structure, bonding, and preliminary reactivity.

The starting material [U(BIPM^TMS^)(Cl)(μ-Cl)_2_Li(THF)_2_] (**1**, BIPM^TMS^=C(PPh_2_NSiMe_3_)_2_)[2j] was treated with two equivalents of benzyl potassium to afford, after workup and recrystallization, the brown uranium(IV) carbene dialkyl complex [U(BIPM^TMS^)(CH_2_Ph)_2_] (**2**) in 72 % yield.[13] Although a number of uranium carbene derivatives have now been reported, dialkyls were unknown.[2] Treatment of **2** with mesitylamine, Scheme 1,[13] gave the uranium(IV) carbene imido complex [{U(BIPM^TMS^)(μ-NMes)}_2_] (**3**) as brown crystals in 92 % yield. We tested the oxidation of **3** with common oxygen-atom-transfer reagents and found that whilst morpholine *N*-oxide, pyridine-*N*-oxide, and trimethylamine-*N*-oxide all gave intractable products, tetramethylpiperidine-*N*-oxide (TEMPO) effected clean oxidation to afford the black uranium(VI) carbene imido oxo complex [{U(BIPM^TMS^)(NMes)(μ-O)}_2_] (**4**) as a crystalline product in 57 % yield. Complex **4** was treated with two equivalents of DMAP to give the uranium(VI) carbene imido oxo complex [U(BIPM^TMS^)(NMes)(O)(DMAP)_2_] (**5**) as black crystals. Complex **5** can also be prepared in 49 % yield from a one-pot reaction of **3** with TEMPO and two equivalents of DMAP.

**Scheme 1:**
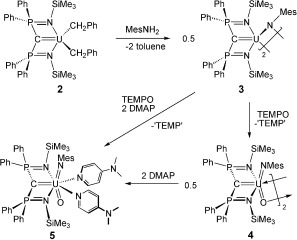
Synthesis of compounds 3–5. (Compound 2 was prepared from 1 and KCH_2_C_6_H_5_).

The characterization data for compounds **2**–**5** are consistent with their formulations. The ^31^P NMR spectrum of **5** has a resonance signal at δ=−22 ppm, shifted from δ=−35 ppm for **4**. Despite exhaustive attempts we could not locate the carbene resonances in the ^13^C NMR spectra of **4** or **5** in the range δ=−200 to +1000 ppm and no folded-in resonances could be detected; 2D ^13^C-^31^P NMR experiments showed only one cross-peak for the *P*-phenyl *ipso* carbon atoms. In contrast, in a cerium(IV) BIPM^TMS^ carbene complex, this method easily located the carbene resonance at δ=+325 ppm.[14] The FTIR spectra of **4** and **5** exhibit strong bands at 837 and 900 cm^−1^, which we attribute to bridging and terminal oxo groups, respectively. The UV/Vis electronic absorption spectra of **2**–**5** are dominated by LMCT (ligand-to-metal charge transfer) absorptions that tail in from the UV region to the visible and the NIR regions are generally featureless (**4** and **5**) or exhibit very weak f→f absorptions (**2** and **3**). The profile of the experimental UV/Vis absorption spectrum of **5** is reproduced well by SAOP/ZORA/TZP TD-DFT calculations, with the absorptions in the *λ*=400–750 nm range arising principally from LMCT transitions involving the carbene and imido lone pairs to vacant uranium 5f orbitals.[13] The uranium(IV) formulations of **2** and **3** were confirmed by SQUID magnetometry.[13] The magnetic moment of **2** is 2.6 μ_B_ at 298 K and this falls to 0.8 μ_B_ at 1.8 K. For **3**, the magnetic moment at 298 K is 3.4 μ_B_ (2.4 μ_B_ per uranium center) and this falls to 1.04 μ_B_ at 1.8 K (0.7 μ_B_ per uranium center). The magnetic moments of **2** and **3** both tend to zero and are consistent with uranium(IV) which is a magnetic singlet at low temperature. We find no evidence of magnetic coupling between the two uranium(IV) centers in **3**, but coupling between uranium(IV) centers is rarely observed.[15]

Compounds **2**–**5** have been characterized by single-crystal X-ray diffraction.[13] The structure of **5** (Figure 1b) confirms the monomeric formulation. In this structure, the uranium center is coordinated to terminal carbene, imido, and oxo groups with two coordinated molecules of DMAP completing a pentagonal-bipyramidal coordination sphere. Notably, the oxo and imido groups adopt axial positions in a T-shaped motif with respect to the carbene. However, unlike uranyl which typically exhibits O-U-O angles of more than 172°, the N-U-O angle is distorted significantly from linearity at 167.14(9)°. This angle is close to the angle of 161° measured in gas-phase UO_3_ which also adopts a distorted T-shaped geometry.[16] The N-U-O angle in **5** is slightly closer to linearity than in **4** (160.49(11)°; Figure 1a), but this most likely reflects the increase in coordination number at uranium in **5** (seven-coordinate) compared to **4** (six-coordinate). This is supported by the significantly different U—N_DMAP_ bond lengths in **5** (2.592(2) and 2.611(2) Å) that are consistent with a more congested coordination environment in **5** compared to **4**. Also, the N-U-O bond angle in **4** may be distorted because of the bridging oxo groups. The U—O bond length in **5** is 1.814(2) Å, which is approximately 0.14 Å shorter than in **4** presumably as a result of its terminal nature. The U—N_imido_ linkage is essentially linear (U-N-C_*ipso*_ ∡=174.2(2)°) and the U—N_imido_ bond length of 1.921(2) Å in **5** is comparable to **4**. The U—O and U—N bond lengths in **5** are each approximately 0.1 Å longer than the analogous distances in [U(N^*t*^Bu)(O)(I)_2_(OPPh_3_)_2_],[8c] perhaps reflecting the presence of the BIPM^TMS^ carbene. The U—C_carbene_ bond length of 2.400(3) Å in **5** is indistinguishable from the analogous bond length in **4** (2.408(3) Å) and is essentially the same as the analogous distances in **2** and **3** (2.351(4) and 2.396(10) Å, respectively).[13] This similarity may reflect the constraints imposed on the carbene by residing in a pincer ligand, but also that with two π-donor ligands already coordinated to uranium this metal ion is electron-rich. A similar effect has been observed in the uranyl(VI) carbene complex [UO_2_{C(PPh_2_S)_2_}(C_5_H_4_N)_2_].[2f] Note that the imido rather than the carbene is *trans* to the oxo in **4** and **5**, an observation which can be rationalized by an ITI effect. For actinyls, the semi-core 6p_*z*_ orbital hybridizes with and transfers charge to 5f orbitals. This transfer leaves a hole in the 6p_*z*_ orbital directed to the *trans* position so that the ligand bonds more strongly to compensate.[11] Taking the oxo as the reference group, the ligand that in principle can donate *trans* electron density most strongly, and hence compensate for the 6p hole the most, is the imido group, which is experimentally observed.

**Figure 1:**
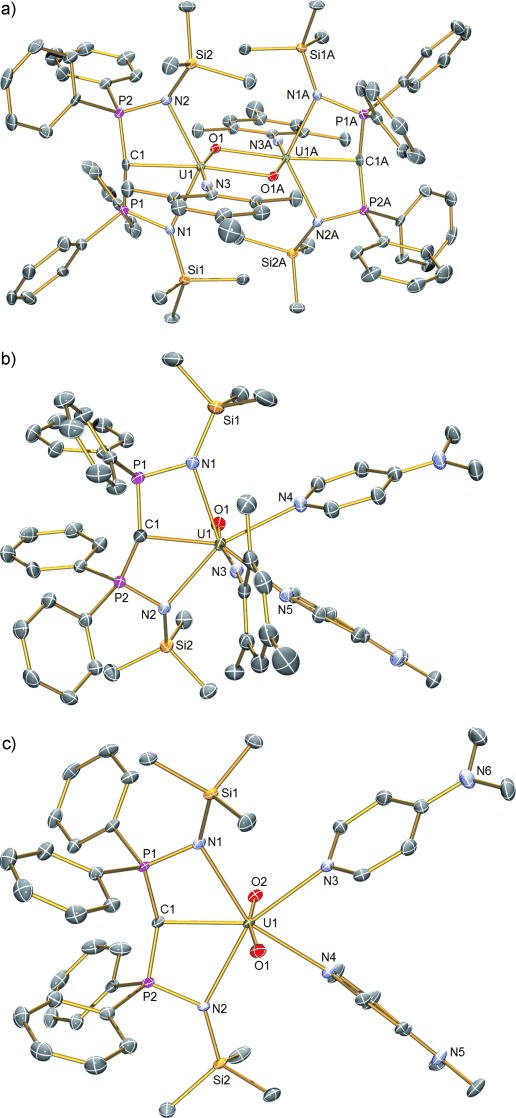
Single-crystal X-ray structures of a) [{U(BIPM^TMS^)(NMes)(μ-O)}_2_] (4), b) [U(BIPM^TMS^)(NMes)(O)(DMAP)_2_] (5), and c) [U(BIPM^TMS^)(O)_2_(DMAP)_2_] (6). Displacement ellipsoids set at 40 % probability. Hydrogen atoms, any lattice solvent, and minor disorder components are omitted for clarity. Selected bond lengths [Å]: 4: U1–C1 2.408(3), U1–N1 2.408(3), U1–N2 2.374(3), U1–N3 1.943(3), U1–O1 1.953(2), U1–O1A 2.337(2); 5: U1–C1 2.400(3), U1–N1 2.554(2), U1–N2 2.577(2), U1–N3 1.921(2), U1–N4 2.592(2), U1–N5 2.611(2), U1–O1 1.814(2); 6: U1–C1 2.383(3), U1–N1 2.606(2), U1–N2 2.600(2), U1–N3 2.564(3), U1–N4 2.594(3), U1–O1 1.794(2), U1–O2 1.785(2).

We conducted DFT calculations on complex **5** which compare well to the experimental solid-state data and we conclude the calculations represent a qualitative model of the electronic structure of **5**. Donation of electron density from the ligands to the uranium center in **5** is suggested by calculated charges of +3.66, −1.91, −1.24, and −0.90 for the uranium, carbene, imido, and oxo centers, respectively. The BIPM^TMS^ P- and N-centers exhibit calculated charges of +1.56 and −1.43, respectively. The calculated charges suggest that the dipolar resonance form of BIPM dominates in this complex.[17] The P—N and P—C_carbene_ Nalewajski–Mrozek (NM) bond indices are calculated as 1.09 and 1.10, respectively. Multiple-bond interactions to uranium from the carbene, imido, and oxo groups are suggested by NM bond indices of 1.23, 2.34, and 2.68, respectively. For comparison, the formally dative imino and pyridine U—N NM bond indices average 0.69 and 0.40, respectively. Uranium BIPM carbenes exhibit NM bond indices in the range 1.2–1.5 for the U—C interaction,[2] and the imido and oxo bond indices are consistent with threefold bonding interactions. Examination of the Kohn–Sham orbitals of **5** reveals a frontier orbital manifold that exhibits σ- and π-interactions involving the carbene, imido, and oxo donors. However, these orbitals are extensively delocalized across each donor group and the uranium center, precluding an assessment of ITI effects; this contrasts to calculations on **6** (see below) where the orbitals are more localized as discrete U—C or [O—U—O]^2+^ combinations.[13]

To develop a more chemically intuitive bonding picture of **5** we examined the uranium carbene, imido, and oxo bonding interactions by natural bond orbital (NBO) analysis.[13] The uranium–carbene σ-bond is composed of 15 % U and 85 % C character. From this σ-bond, the uranium component contains 0.4 % 7s-, 0.3 % 7p-, 19.6 % 6d-, and 79.7 % 5f-orbital contributions whereas the carbon component is composed of 16.6 % 2s- and 83.4 % 2p-orbital contributions. The uranium–carbene π-bond is composed of 18.4 % U and 81.6 % C contributions. The carbon component of this bond is essentially 100 % 2p-orbital hybridized, reflecting the π-character of this orbital, whereas the uranium component comprises 0.4 % 7s-, 0.2 % 7p-, 5.5 % 6d-, and 93.9 % 5f-orbital contributions. The two uranium–imido π-bonds are essentially identical and are composed of 23.3 % U and 76.7 % N contributions. The uranium component comprises 11.7 % 6d-and 88.3 % 5f-orbital contributions with no 7s or 7p components whereas the nitrogen component comprises essentially 100 % 2p-orbital character, in agreement with the π-bonding nature of these orbitals. No formal U—N_imido_ σ-bond was indicated by the NBO calculations. The U—O π-bonds are returned as being primarily localized on the oxygen whereas the U—O σ-bond is identified by NBO as being composed of 23.3 % U and 76.7 % O character. The uranium component has 1.5 % 7s-, 0.3 % 7p-, 9.3 % 6d-, and 88.9 % 5f-orbital character whereas the oxygen contributions are 12.7 % 2s and 87.3 % 2p. The calculations suggest that uranium principally employs 5f rather than 6d orbitals in the multiple bonds to the carbene, imido, and oxo centers in **5** as has been determined in other uranium–ligand multiple bonds.[2–4]

To provide a topological analysis of the UL interactions in **5**, we used Bader’s quantum theory of atoms in molecules (QTAIM). In QTAIM, a chemical bond is defined by the presence of a line of locally maximum electron density [*ρ*(**r**)] along a bond path between two atoms and by a bond critical point (BCP) representing the minimum in the electron density along the locally maximal line. For a covalent bond, *ρ*(**r**) at the BCP between two nuclei is usually greater than 0.1 and the electronic energy-density term *H*(**r**) is usually negative for a covalent bond. The calculated *ρ*(**r**) and *H*(**r**) values for the U—C, U—N, and U—O 3,−1 BCPs are 0.092/−0.031, 0.185/−0.109, and 0.247/−0.186, respectively. The corresponding values for the uranium–imino and uranium–pyridine dative bonds average 0.0478/−0.004, respectively. The ellipticity of a BCP provides quantification of the σ/π character of a bond; for a σ- or σ-/2π-bond, which present cylindrical contours of electron density, the ellipticity is approximately 0, and for a σ-/π-bond the ellipticity is greater than 0 arising from the asymmetric electron-density distribution which is perpendicular to the bond path. The ellipticities of ethane, benzene, ethene, and acetylene are calculated to be 0.00, 0.23, 0.45, and 0.00, respectively.[18] Group 6 carbonyl complexes exhibit ellipticities of approximately 0,[19] whereas M—C interactions exhibit ellipticities in the range 0.20–0.62.[20] The calculated ellipticity for the U—C bond in **5** (0.21) is comparable to the C—C bonds in benzene. For the U—C interactions in complexes [U(BIPM^TMS^H)(Cl)_3_(THF)],[2e] [U(BIPM^TMS^)(I)_2_(Cl)],[2j] [UOCl_2_(BIPM^TMS^)],[2k] and [U(C_5_H_5_)_3_C(H)PMe_3_],[21] we previously calculated ellipticities of 0.04, 0.35, 0.38, and 0.26, respectively. Where only a spherical σ-bonding interaction is possible in the first of this series the ellipticity is approximately 0, but for the remaining complexes the ellipticities are similar to those calculated for **5**. The U—N bond ellipticity (0.11) in **5** is smaller than the U—C interaction and its deviation from zero most likely reflects conjugative effects to the N-aryl ring.[18] The ellipticity for the uranium–oxo bond (0.03) suggests a triple-bond interaction.[18]

Whilst we note that dipolar U^+^—L^−^ resonance structures will contribute to the overall bonding picture of the uranium–ligand multiple bonds in **5**, the combined computational data are in agreement; all identify multiple bond combinations for all of the uranium–carbene, -imido, and -oxo linkages that are polarized but which involve more than one electron-pair per heteroatom and are thus multiple in nature.

Our preliminary investigations on the reactivity of complex **5** have shown it to be reactive. Complex **5** was allowed to react with *tert*-butylisocyanate to afford the uranyl carbene complex [U(BIPM^TMS^)(O)_2_(DMAP)_2_] (**6**; Figure 1c) ^[13]^ as black crystals in 67 % yield with concomitant elimination of *tert*-butylmesitylcarbodiimide (Scheme 2).[13] The identity of the carbodiimide by-product was confirmed by comparison of the NMR spectra to literature data.[22] Although the resonance signals in the ^31^P NMR spectra of **5** and **6** are within 0.3 ppm of each other (δ≈−22 ppm), the reaction of **5** with *tert*-butylisocyanate proceeds via an intermediate that we could not isolate. This intermediate exhibits a ^31^P NMR resonance at δ=−44 ppm which suggests the formation of a [2+2]-cycloaddition product.[6d]It is germane to note that all previous attempts to prepare complex **6**, by deprotonation of [UO_2_(Cl)(BIPM^TMS^H)(THF)][23] with a wide range of bases, or oxidation of carbene precursors, failed and instead afforded pentavalent or hexavalent uranyl methanides.[2k,l]

**Scheme 2:**
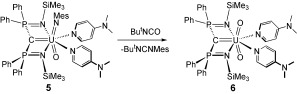
Synthesis of complex 6 from complex 5.

To conclude, by installing carbene, imido, and oxo groups at a uranium center by salt elimination, protonolysis, and two-electron oxidation, it has been possible to prepare a complex with three formal covalent multiply bonded ligands where the coordinated heteroatoms derive from different element groups. Computational analyses suggest formal U—C double bond and triple-bonding interactions for the imido and oxo linkages. In all cases, the computational data suggest the dominance of uranium 5f rather than 6d orbitals in the three multiple bonds. The delocalization of the frontier orbitals involved in the uranium–carbene, -imido, and -oxo interactions suggests that the intuitive formulation of **5** as a carbene N—U—O uranyl analogue is not appropriate. This conclusion is also consistent with the preliminary reactivity study of **5** which has enabled the preparation of a previously inaccessible uranyl carbene complex through N for O metathesis reactivity at the imido group,[3o, 6d] rather than at the carbene.
