# Supplementary material for: Synthesis, Characterization, and Reactivity of a Uranium(VI) Carbene Imido Oxo Complex
Source: Angew Chem Int Ed Engl. 2014 May 19;53(26):6696–700. doi: 10.1002/anie.201403892 (PMC4464547; doi:10.1002/anie.201403892)
Supplement: Supplementary file 1 [file anie0053-6696-sd1.pdf]

Angewandte Chemie
Eine Zeitschrift der Gesellschaft Deutscher Chemiker

Supporting Information
© Wiley-VCH 2014
69451 Weinheim, Germany

Synthesis, Characterization, and Reactivity of a Uranium(VI) Carbene Imido Oxo Complex**

*Erli Lu, Oliver J. Cooper, Jonathan McMaster, Floriana Tuna, Eric J. L. McInnes, William Lewis, Alexander J. Blake, and Stephen T. Liddle**

anie_201403892_sm_miscellaneous_information.pdf

Experimental

General

All manipulations were carried out using Schlenk techniques, or an MBraun UniLab glovebox, under an atmosphere of dry nitrogen. Solvents were dried by passage through activated alumina towers and degassed before use. All solvents were stored over potassium mirrors except for ethers which were stored over activated 4 Å sieves. Deuterated solvent was distilled from potassium, degassed by three freeze-pump-thaw cycles and stored under nitrogen. [U(BIPM^{TMS})(Cl)(μ-Cl)$_2$Li(THF)$_2$] [**1**, BIPM^{TMS} = C(PPh$_2$NSiMe$_3$)$_2$] and [KCH$_2$Ph] were prepared as described previously.[1,2] *N*-oxides, DMAP, *tert*-butylisocyanate, and benzaldehyde were variously dried under vacuum or over activated 4 Å sieves for 4 hours, either neat or dissolved in ethers, prior to use. Bu^{t}NCNMes byproduct from the preparation of **6** was identified by comparison of its NMR spectra with literature data.[3]

^{1}H, ^{13}C, ^{29}Si, and ^{31}P, NMR spectra were recorded on a Bruker 400 spectrometer operating at 400.2, 100.6, 79.5, and 162.0 MHz respectively; chemical shifts are quoted in ppm and are relative to TMS (^{1}H, ^{13}C, ^{29}Si) and external 85% H_3PO_4 (^{31}P). FTIR spectra were recorded on a Bruker Tensor 27 spectrometer. UV/Vis/NIR spectra were recorded on a Perkin Elmer Lambda 750 spectrometer. Data were collected in 1mm path length cuvettes loaded in an MBraun UniLab glovebox and were run versus the appropriate THF reference solvent. Variable-temperature magnetic moment data were recorded in an applied dc field of 0.1 T on a Quantum Design MPMS XL7 superconducting quantum interference device (SQUID) magnetometer using doubly recrystallized powdered samples. Care was taken to ensure complete thermalisation of the sample before each data point was measured and samples were immobilised in an eicosane matrix to prevent sample reorientation during measurements. Diamagnetic corrections of 488.5×10^{-6} and 965.03×10^{-6} cm^3 mol^{-1} were applied for **2** and **3**

using tabulated Pascal constants and measurements were corrected for the effect of the blank sample holders (flame sealed Wilmad NMR tube and straw) and eicosane matrix. Elemental microanalyses were carried out by Tong Liu at the University of Nottingham.

Preparation of [U(BIPM^{TMS})(CH$_2$Ph)$_2$] (2)

THF (10 ml) was added to a pre-cooled (−78 °C) mixture of **1** (1.09 g, 1.0 mmol) and [KCH$_2$Ph] (0.13g, 2.0 mmol). The mixture was then allowed to slowly warm to room temperature with stirring over 6 hours to afford a dark red solution. Volatiles were removed *in vacuo* and then extracted with toluene. The mixture was reduced in volume (~3 ml) then stored at −30 °C to afford **2** as brown crystals. Yield 0.70 g, 72%. Anal Calcd for $C_{45}H_{52}N_2P_2Si_2U$: C, 55.31; H, 5.36; N, 2.86. Found: C, 54.46; H, 5.27; N, 2.90. ^{1}H NMR (C_6D_6, 298 K): δ −39.09 (s, 4H), −15.68 (s, 18H, Si(C*H*$_3$)$_3$), 0.23 (s, 2H, *p*-Ar-C*H*), 3.89 (s, 4H), 8.00 (s, 4H), 8.97 (s, 8H, Ph-C*H*), 16.81 (s, 8H, Ph-C*H*), 29.85 (s, br, 4H). ^{31}P{^{1}H} NMR (C_6D_6, 298 K): *δ* −427.8 (s, br). FTIR *ν*/cm^{-1} (Nujol): 1653 (w), 1437 (m), 1307 (m), 1283 (m), 1175 (w), 1107 (s), 1007 (br).

Preparation of [{U(BIPM^{TMS})(μ-NMes)}$_2$] (3)

A solution of 2,4,6-trimethylaniline (0.27 g, 2.0 mmol) in toluene (5 ml) was added to a solution of **2** (1.95 g, 2.0 mmol) in toluene (5 ml) at –78 °C. The dark red mixture was stirred at –78 °C for 15 minutes, then was allowed to warm to ambient temperature with stirring for 4 h. The brown solution was filtered and volatiles were removed *in vacuo* to afford the product as a brown solid. Yield: 1.72 g, 92 %. Single crystals of **3** suitable for X-ray single crystal diffraction study were obtained from toluene solution at –30 °C. Anal. Calcd. for $C_{80}H_{98}N_6P_4Si_4U_2.C_6H_5CH_3$: C, 53.95; H, 5.40; N, 4.29%. Found: C, 53.34; H, 5.54; N, 4.21. ^{1}H NMR (C_6D_6, 298 K): *δ* –23.14 (s, 36H, Si(C*H$_3$*)*$_3$*), 0.27 (s, 6H, *para*-C*H$_3$* of Mes), 0.30 (s,

12H, *ortho*-CH_3 of Mes), 8.39 (br, 6H, Ar*H*), 9.01 (s, br, 10H, Ar*H*), 10.24 (s, br, 16H, Ar*H*), 24.13 (br, 12H, Ar*H*). $^{31}P\{^1H\}$ NMR (C_6D_6, 298 K): δ 38.12 (s, br). FTIR v/cm^{-1} (Nujol): 1299 (m), 1203 (s), 1148 (s), 1105 (s), 1016 (s), 835(m), 771 (m), 717 (m), 694 (m), 656 (w), 605 (s), 585 (s), 557 (m), 503 (m), 431 (m).

Preparation of [{U(BIPM^{TMS})(NMes)(μ-O)}$_2$] (4)

A solution of TEMPO (0.06 g, 0.41 mmol) in toluene (2 ml) was added to a solution of **3** (0.38 g, 0.21 mmol) in toluene (3 ml) at –78 °C. The black mixture was stirred at –78 °C for 5 minutes, then was allowed to warm to ambient temperature with stirring for 16 h. The black solution was filtered and concentrated to approximately 1 ml. After storing at –35 °C overnight, **4** was obtained as a black crystalline solid. Yield: 0.22 g, 57 %. Single crystals which were suitable for X-ray single crystal diffraction study were obtained from d_6-benzene solution at ambient temperature. Anal. Calcd. for $C_{80}H_{98}N_6O_2P_4Si_4U_2$: C, 50.89; H, 5.23; N, 4.45. Found: C, 50.94; H, 5.57; N, 4.69. ^1H NMR (C_6D_6, 298 K): δ 0.53 (s, 36H, Si(CH_3)$_3$), 4.12 (s, 6H, *para*-CH_3 of Mes), 4.21 (br, 12H, *ortho*-CH_3 of Mes), 7.03 – 7.13 (m, 10H, Ar*H*), 7.21 – 7.25 (m, 6H, Ar*H*), 7.35 – 7.40 (m, 8H, Ar*H*), 7.46 (s, 4H, Ar*H* of Mes), 7.75 – 7.81 (m, 8H, Ar*H*), 8.37 – 8.44 (m, 8H, Ar*H*). ^{13}C NMR (C_6D_6, 298 K): δ 5.15 (s, Si(*C*H_3)$_3$), 16.26 (s, *para*-*C*H_3 of Mes), 16.37 (s, *ortho*-*C*H_3 of Mes), 130.04, 130.18, 130.60, 131.40 (s, ArC), 131.71 (t, $^3J_{PC}$ = 6.1 Hz, *meta*-*C* of P–Ph), 133.40 (t, $^3J_{PC}$ = 6.1 Hz, *meta*-*C* of P–Ph), 134.48 (t, $^3J_{PC}$ = 6.0 Hz, *meta*-*C* of P–Ph), 143.41 (s, ArC). $^{31}P\{^1H\}$ NMR (C_6D_6, 298 K): δ –34.64 (s). $^{29}Si\{^1H\}$ NMR is unavailable due to poor solubility of the compound in C_6D_6 which also accounts for the fact not all ^{13}C resonances, especially in the aromatic region, could be observed. FTIR v/cm^{-1} (Nujol): 1297 (w), 1260 (w), 837 (s) (U=O), 694 (s), 654(w).

Preparation of [U(BIPM^{TMS})(NMes)(O)(DMAP)$_2$] (5)

A mixture of DMAP (0.12 g, 1.0 mmol) and TEMPO (0.08 g, 0.5 mmol) in toluene (5 ml) was added to a solution **3** (0.46 g, 0.25 mmol) in toluene (5 ml) at 0 ^{o}C. The black mixture was stirred at 0 ^{o}C for 10 minutes, then was allowed to warm to ambient temperature with stirring for 1 h. The black mixture was filtered and concentrated to approximately 1 ml. After storing at –35 ^{o}C overnight, **5** was obtained as black crystals. Yield: 0.28 g, 49 %. Single crystals which were suitable for X-ray single crystal diffraction study were obtained from toluene solution at ambient temperature. Anal. Calcd. for $C_{54}H_{69}N_7OP_2Si_2U.1.5(C_6H_5CH_3)$: C, 58.40; H, 6.15; N, 7.39. Found: C, 58.21; H, 6.12; N, 7.08. ^{1}H NMR (C_6D_6, 298 K): δ 0.52 (s, 18H, Si(C*H$_3$*)$_3$), 2.09 (br, 12H, N(C*H$_3$*)$_2$), 2.11 (s, 7.5H, C*H$_3$* of toluene), 2.85 (s, 3H, *para*-C*H$_3$* of Mes), 3.14 (s, 6H, *ortho*-C*H$_3$* of Mes), 6.00 (br, 4H, *meta-H* of DMAP), 6.90 – 6.98 (m, 6H, Ar*H*), 6.99 – 7.14 (m, 7H, Ar*H*), 7.18 – 7.22 (m, 6H, Ar*H*), 7.78 – 7.85 (m, 4H, Ar*H*), 8.15 – 8.23 (m, 4H, Ar*H*), 9.16 (br, 4H, *ortho-H* of DMAP). ^{13}C NMR (C_6D_6, 298 K): δ 4.33 (s, Si(*C*H$_3$)$_3$), 19.20, 19.26 (s, *C*H$_3$ of Mes), 21.10 (s, *C*H$_3$ of toluene), 38.11 (s, N(*C*H$_3$)$_2$ of DMAP), 106.66 (s, *ortho-C* of DMAP), 124.21 (s, Ar*C*), 125.64 (s, *para-C* of toluene), 128.51 (s, *meta-C* of toluene), 129.19 (s, Ar*C*), 129.29 (s, *ortho-C* of toluene), 129.75, 130.61, 131.73, 132.26, 133.32 (s, Ar*C*), 137.84 (s, *ipso-C* of toluene), 140.09 (s, Ar*C*), 143.04, 143.54 (s, Ar*C*), 143.92 (d, J_{PC} = 42.9 Hz, *ipso-C* of P–Ph), 144.35 (d, J_{PC} = 42.8 Hz, *ipso-C* of P–Ph), 152.65 (br, *ortho-C* of DMAP), 154.23, 154.42 (s, Ar*C*). ^{31}P{^{1}H} NMR (C_6D_6, 298 K): δ –22.0 (s). ^{29}Si{^{1}H} NMR (C_6D_6, 298 K): δ –8.28 (t, $^{2}J_{PSi}$ = 5.25 Hz, P=N(*Si*Me$_3$)). FTIR v/cm^{-1} (Nujol): 1959 (w), 1607 (s), 1526 (m), 1288 (m), 1100 (m), 1081 (s), 1064 (s), 900 (m) (U=O), 854 (m), 694 (w), 642 (w), 601 (w), 545 (w), 507 (w).

Preparation of [U(BIPM^{TMS})(O)$_2$(DMAP)$_2$] (6)

A solution of *tert*-butyl isocyanate (0.025 g, 0.26 mmol) in toluene (3 ml) was added to a solution of **5** (0.30 g, 0.26 mmol) in toluene (3 ml) at ambient temperature with stirring. The black mixture was stirred at 50 °C for 15 h and filtered. The black filtrate was concentrated to approximately 1 ml. After storage at −35 °C for 1 day, the product was obtained as a black solid. Yield: 0.18 g, 67%. Single crystals suitable for X-ray diffraction studies were obtained from d_6-benzene solution at ambient temperature. Anal. Calcd. for $C_{45}H_{58}N_6O_2P_2Si_2U.C_6D_6$: C, 50.46; H, 5.46; N, 7.85. Found: C, 50.20; H, 5.47; N, 7.97. All assigments of NMR data are based on *in situ* NMR monitoring of the reaction. This is due to the fact that once **6** is isolated in crystalline form it cannot be redissolved in non-coordinating solvents and dissolution in coordinating solvents results in decomposition. Therefore, ^{13}C and $^{29}Si\{^1H\}$ NMR data are unavailable. ^1H NMR (C_6D_6, 298 K): δ 0.90 (s, 18H, Si*Me$_3$*), 2.08 (s, br, 12H, -N*Me$_2$* of DMAP), 6.07 (br, 4H, *meta-H* of DMAP), 7.00 – 7.05 (m, 16H, Ar*H*), 8.10 – 8.15 (m, 4H, Ar*H*), 8.93 (br, 4H, *ortho-H* of DMAP). $^{31}P\{^1H\}$ NMR (C_6D_6, 121 MHz, 298 K): δ (ppm) = −22.3 (s). FTIR v/cm^{-1} (Nujol): 1613 (s), 1535 (m), 1345 (m), 1240 (w), 1074 (s), 860 (s, U=O), 722 (m), 634(w).

NMR Spectra of 2-5

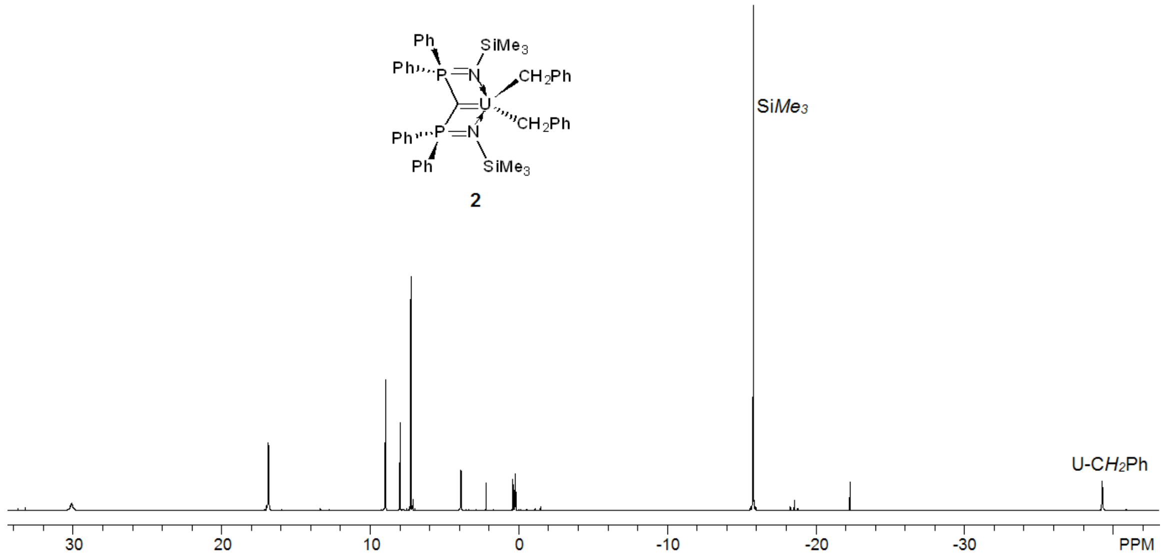

Figure S1. ***^{1}H NMR of 2 (C_6D_6, 25 ^{o}C)***

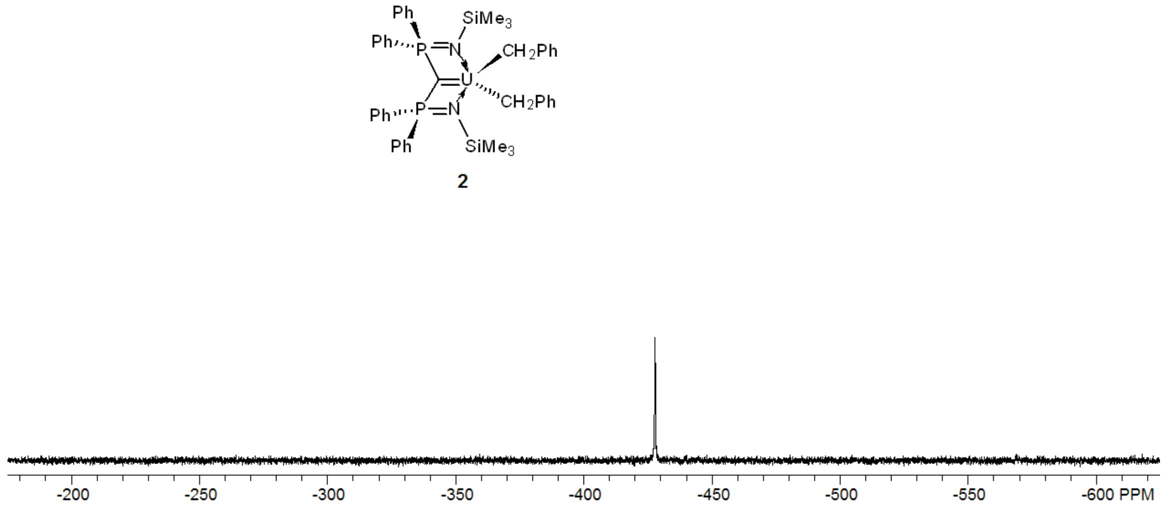

Figure S2. ***^{31}P NMR of 2 (C_6D_6, 25 ^{o}C)***

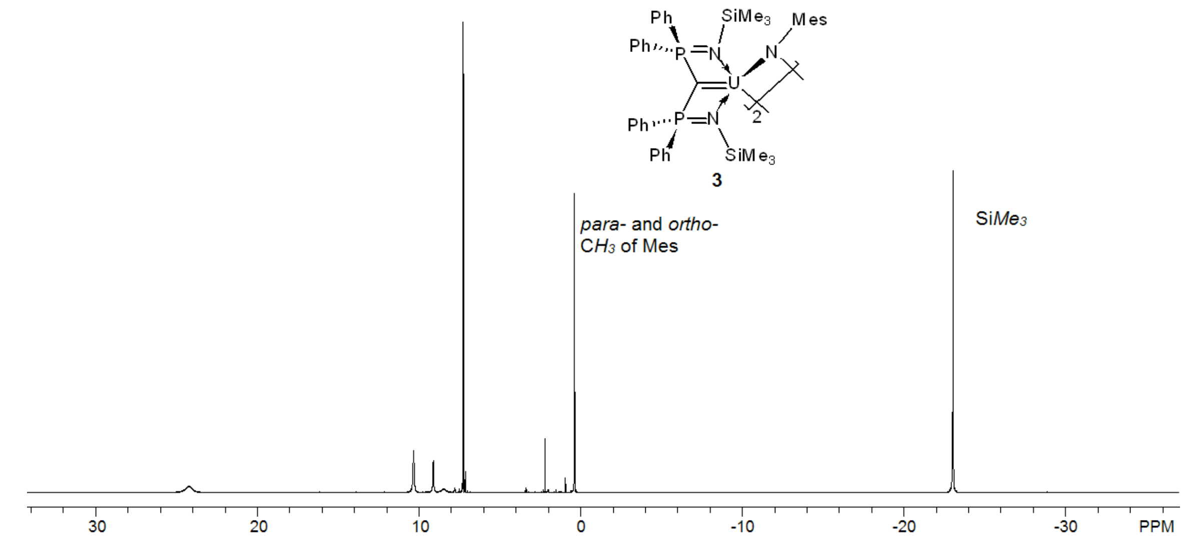

Figure S3. ***^{1}H NMR of 3 (C_6D_6, 25 °C)***

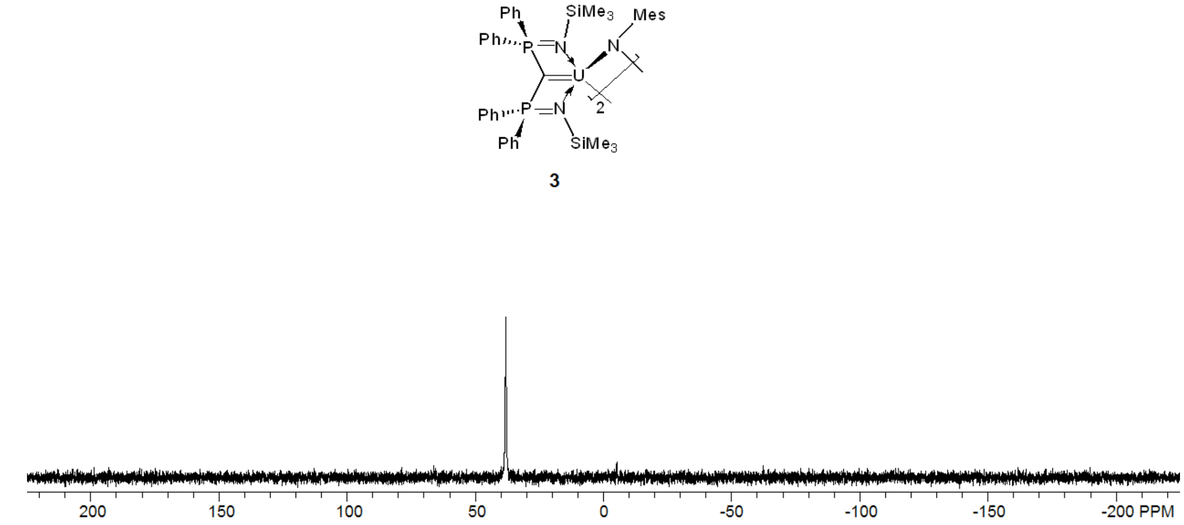

Figure S4. ***^{31}P NMR of 3 (C_6D_6, 25 °C)***

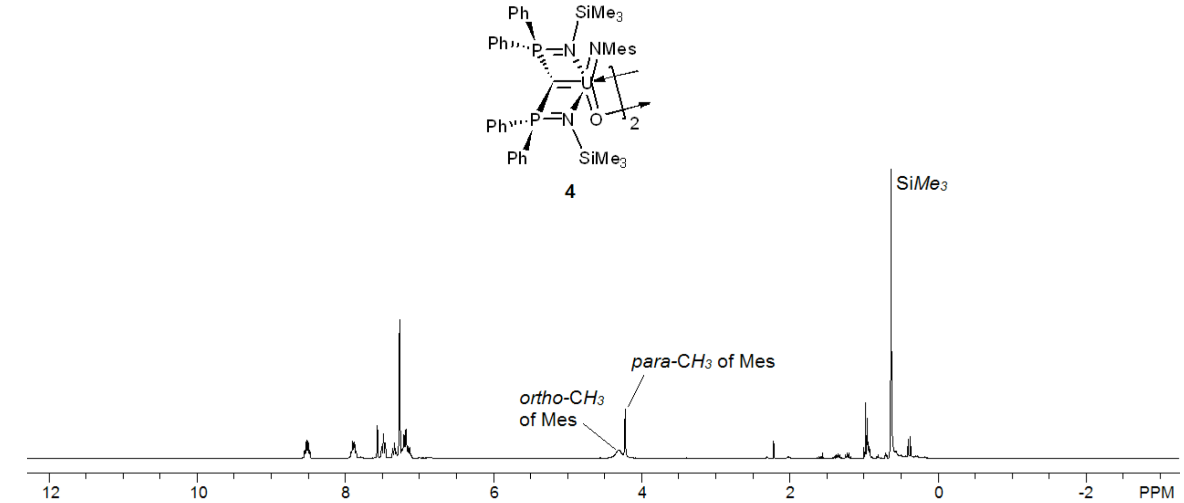

Figure S5. ***^{1}H NMR of 4 (C_6D_6, 25 °C)***

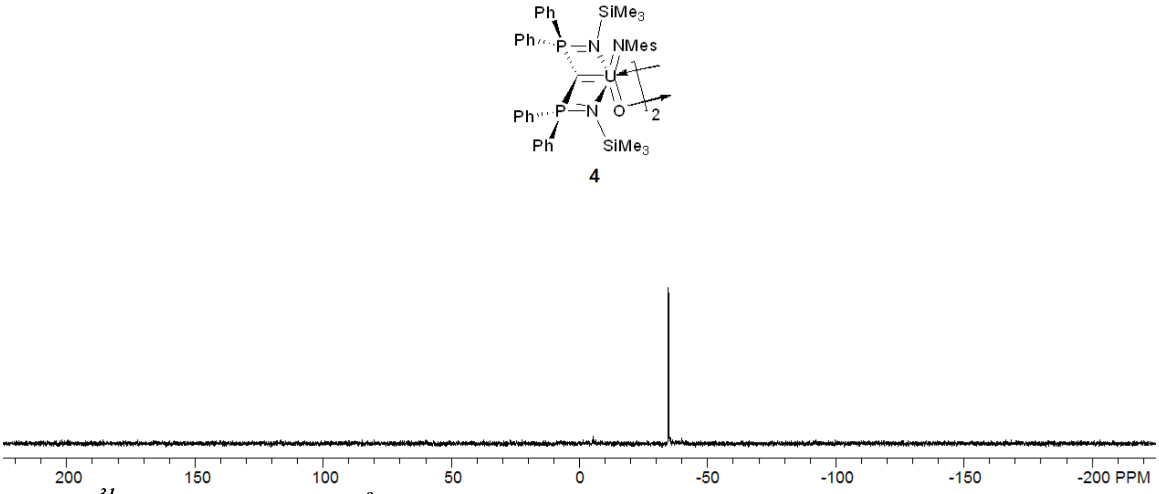

Figure S6. ^{31}***P NMR of 4 (C_6D_6, 25 ^oC)***

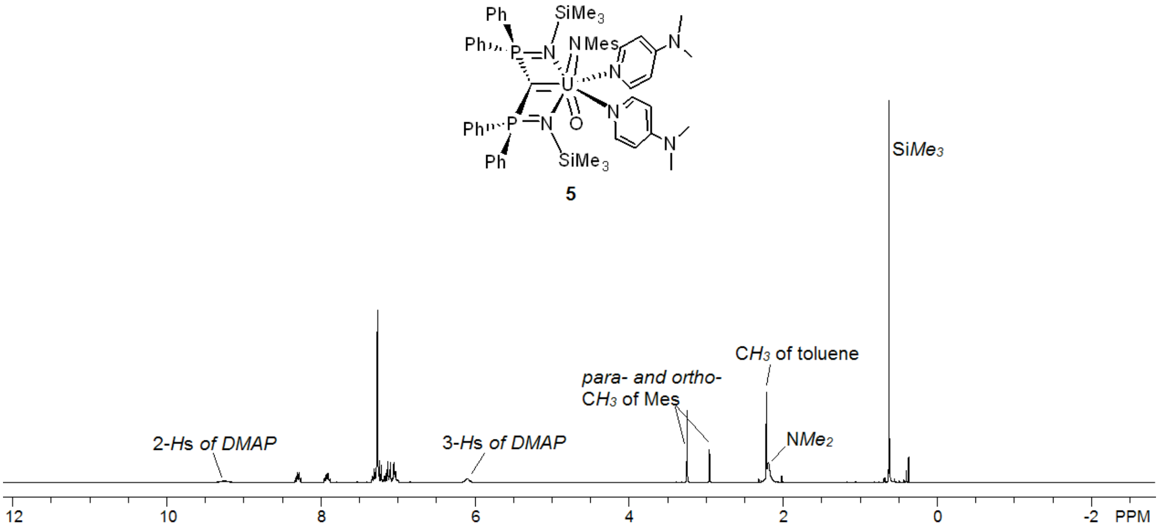

Figure S7. ^{1}***H NMR of 5 (C_6D_6, 25 ^oC)***

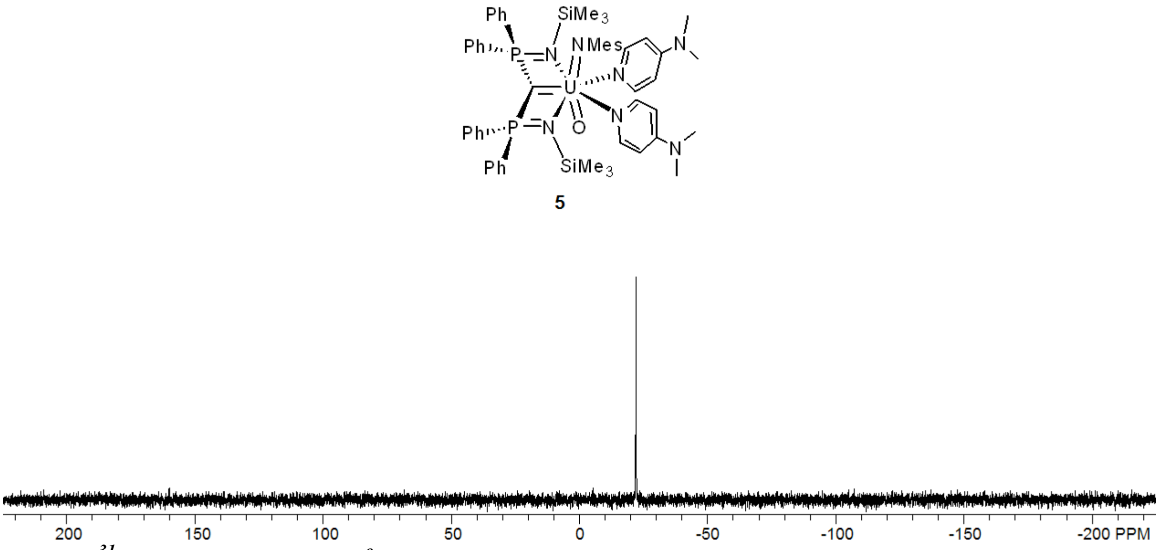

Figure S8. ^{31}***P NMR of 5 (C_6D_6, 25 ^oC)***

^{31}P NMR spectroscopic Monitoring of the Reactivity of 5

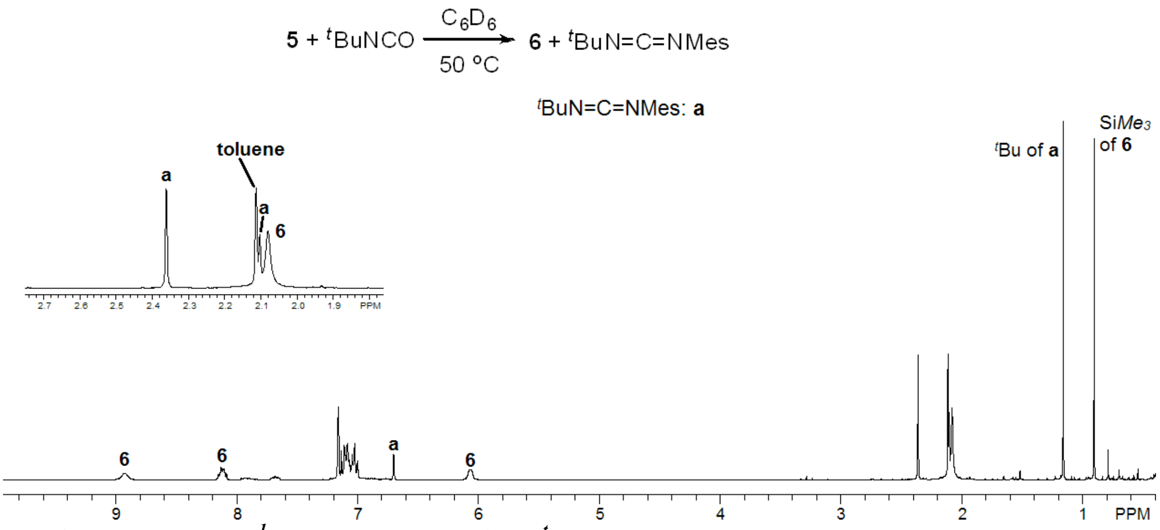

Figure 9. Assignments of ^{1}H NMR signals of 6 and ^{t}BuN=C=NMes based on in situ NMR scale reaction.

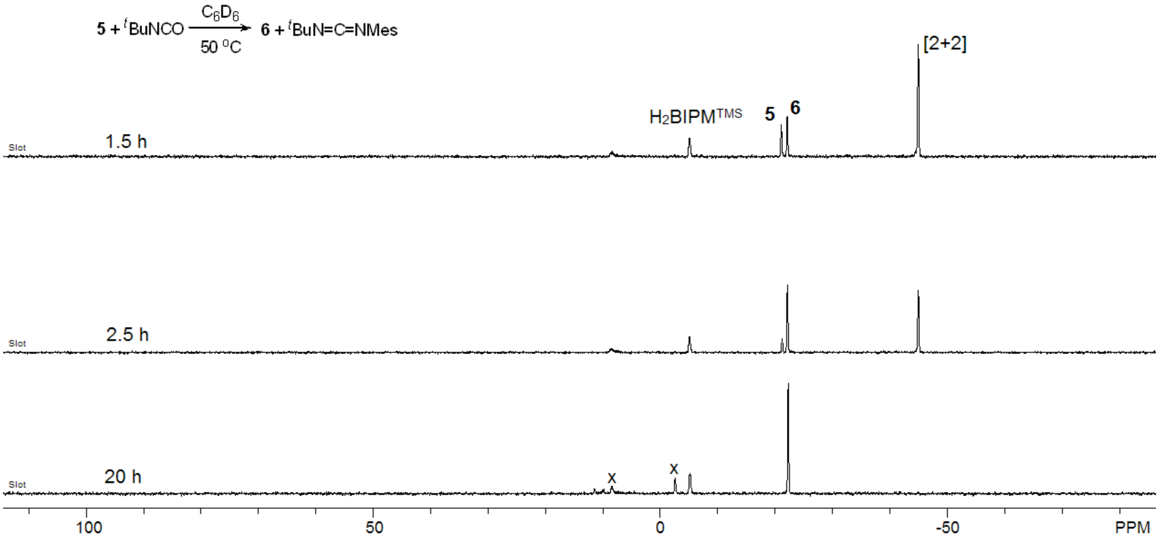

Figure S10. ^{31}P NMR in situ monitoring of the NMR scale reaction between 5 and ^{t}BuNCO. [2+2] denotes the proposed cycloaddition intermediate. x denotes minor unknown components. A minor quantity of free ligand (H_2BIPM^{TMS}) exists as side product of complex decomposition.

Variable Temperature SQUID Measurements

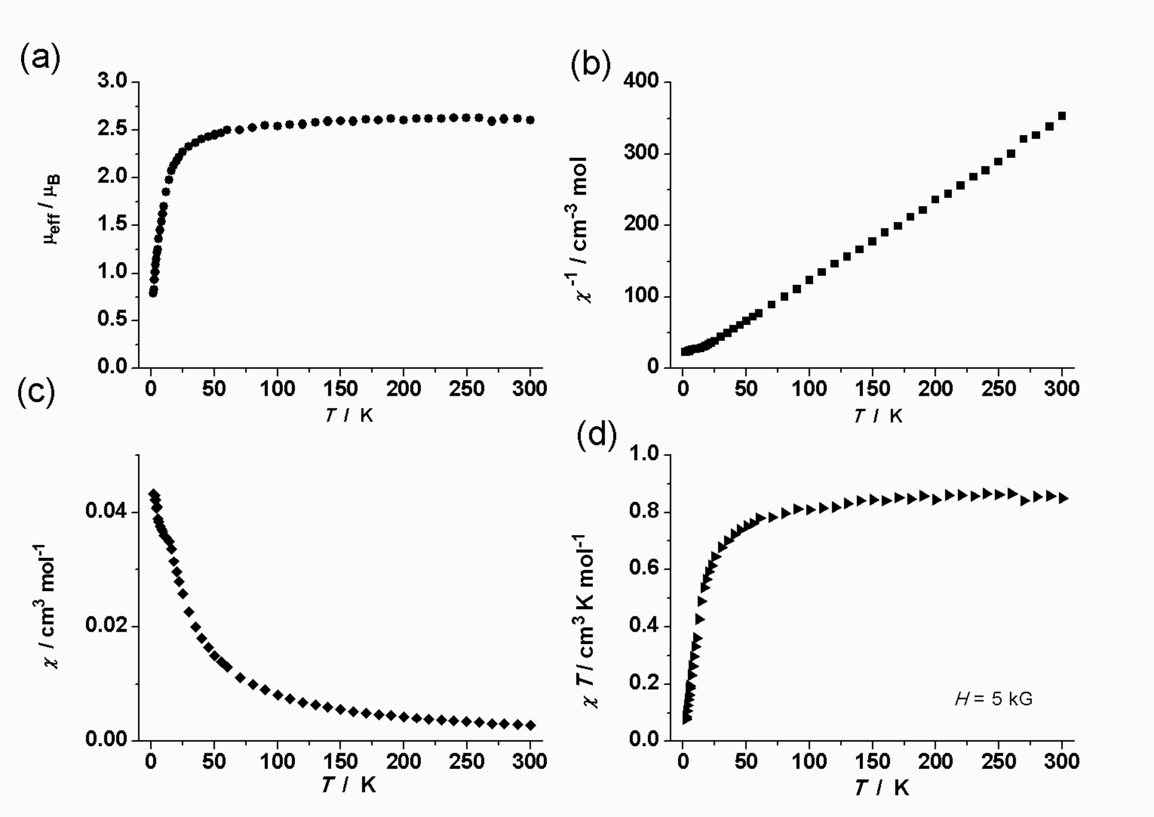

Figure S10. SQUID data for 2: (a) μ_{eff} vs T, (b) $1/\chi$ vs T, (c) χ vs T, (d) χT vs T

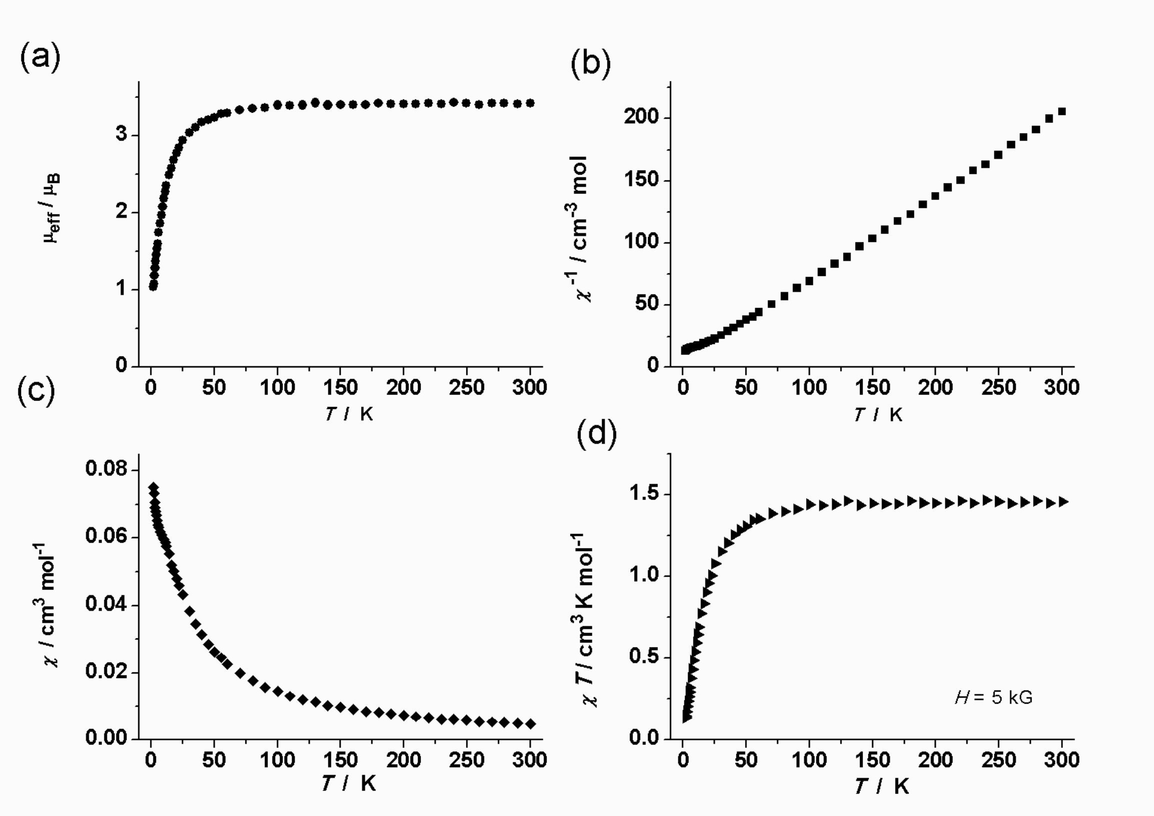

Figure S11. SQUID data for 3: (a) μ_{eff} vs T, (b) $1/\chi$ vs T, (c) χ vs T, (d) χT vs T

Electronic Absorption Spectra

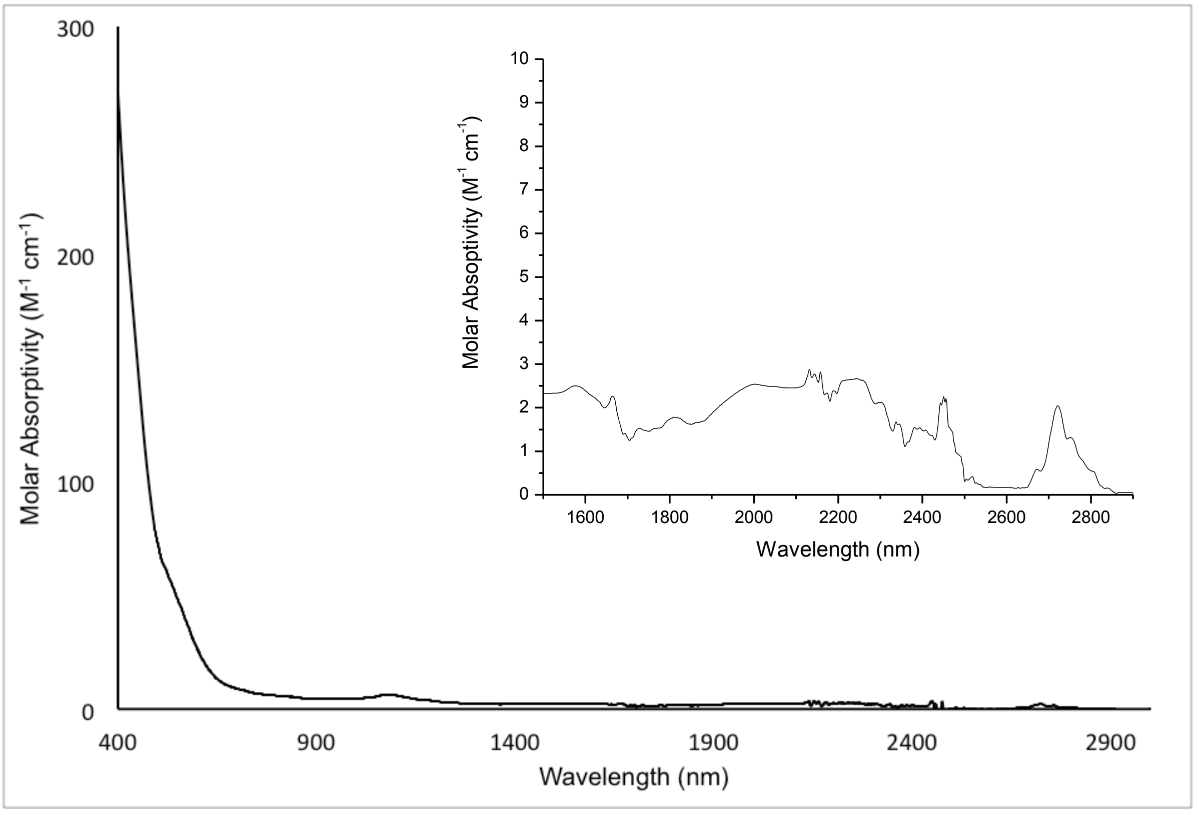

Figure S12. Electronic absorption spectrum of 2, Inset: 1500-2900 nm range

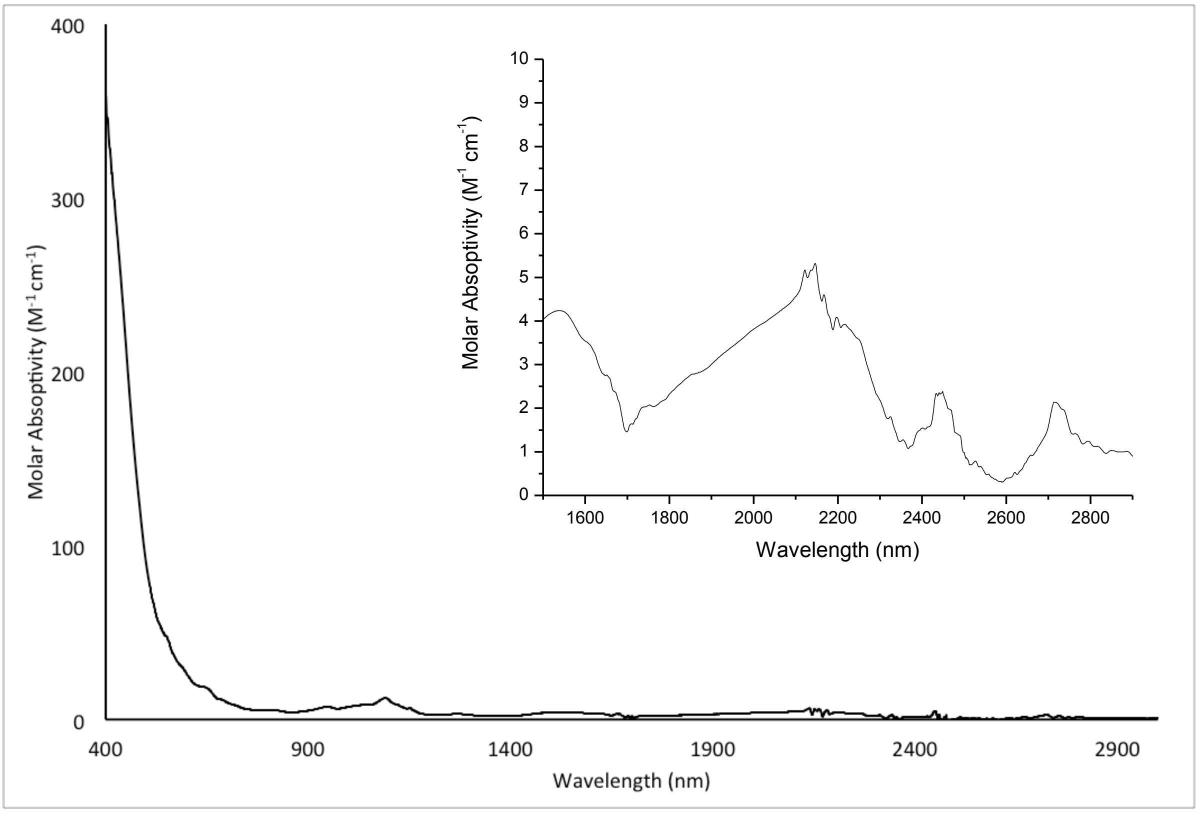

Figure S13. Electronic absorption spectrum of 3, Inset: 1500-2900 nm range

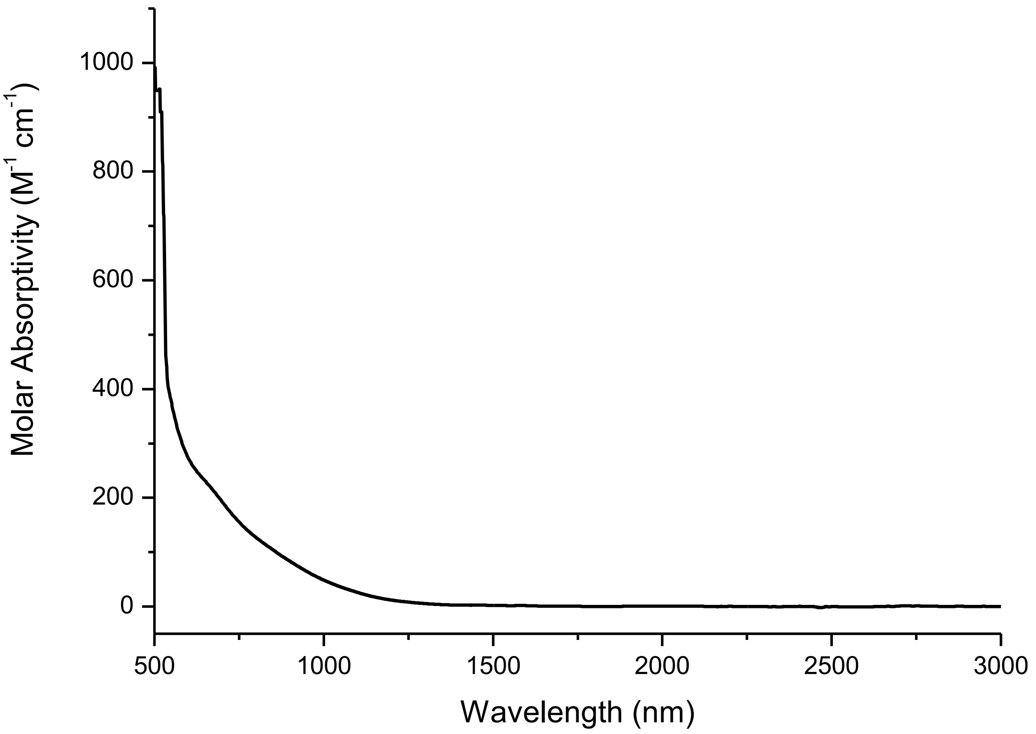

Figure S14. Electronic absorption spectrum of 4

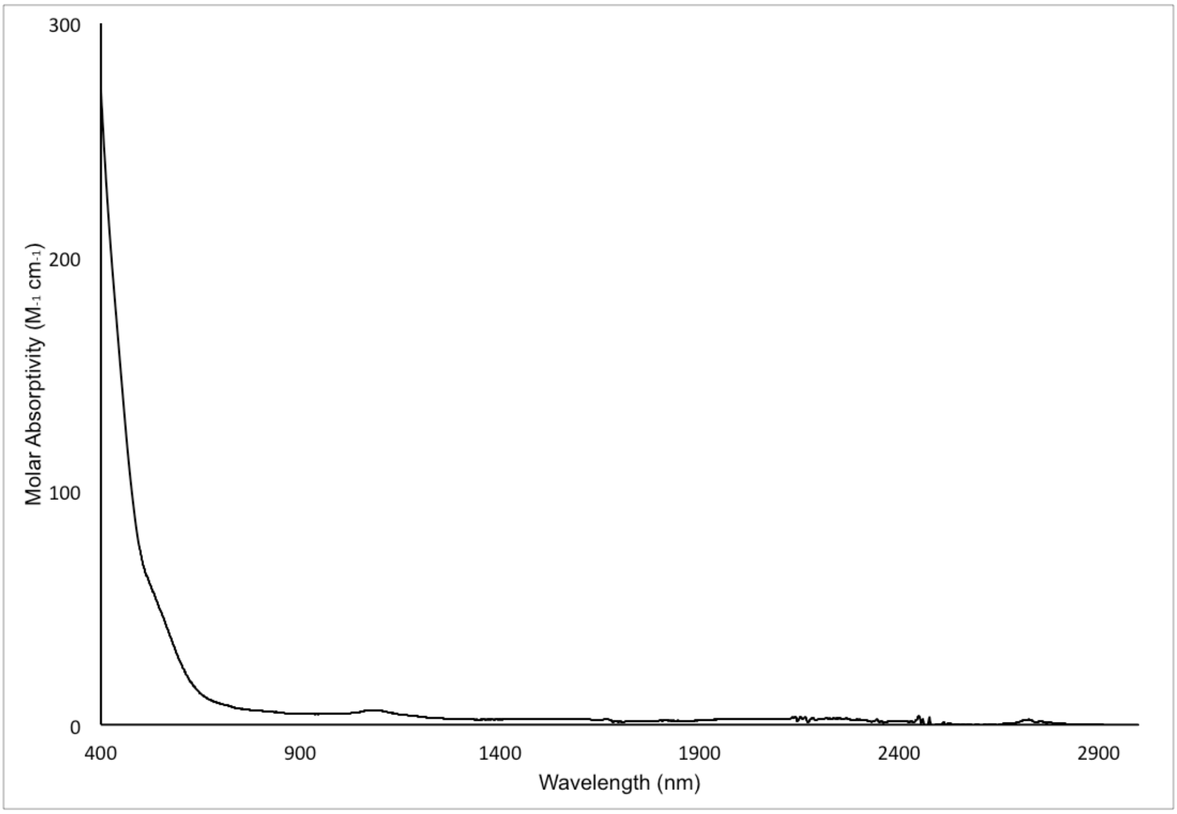

Figure S15. Electronic absorption spectrum of 5

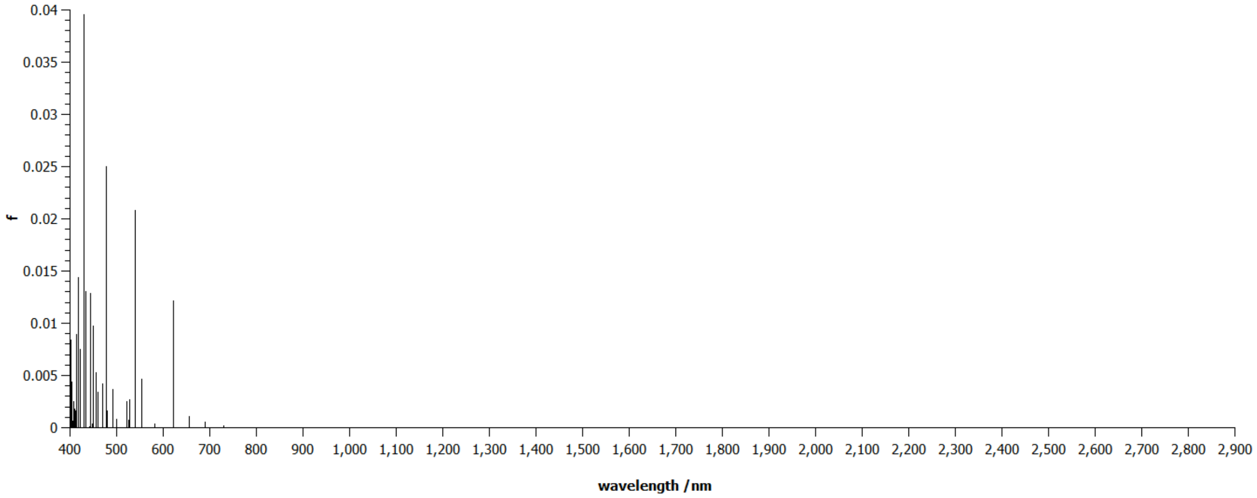

Figure S16. Simulated TD-DFT electronic absorption spectrum of 5 with calculated oscillator strengths depicted as vertical black lines. The transitions correspond to electronic absorptions involving the π-components of the U=C and U=N bonds to vacant 5f-orbitals.

The electronic absorption spectrum of **6** could not be determined because once isolated this complex is essentially insoluble in non-coordinating solvents and it decomposes in polar solvents.

Crystal Structures

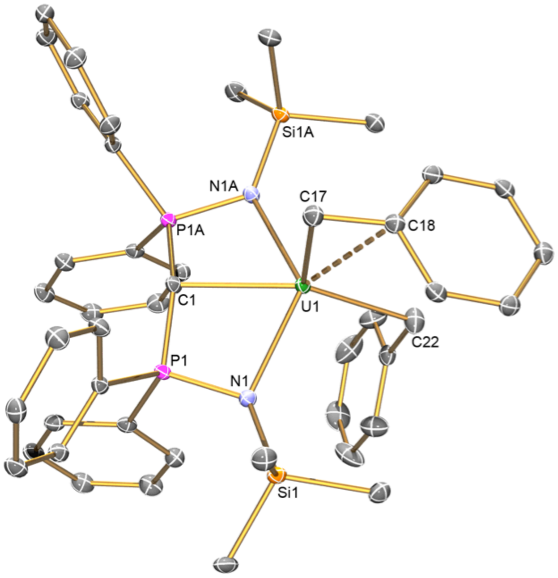

Figure S17. Molecular structure of 2 at 90 K with selective labelling and displacement ellipsoids set to 40%. Lattice solvent molecules and hydrogen atoms omitted for clarity.

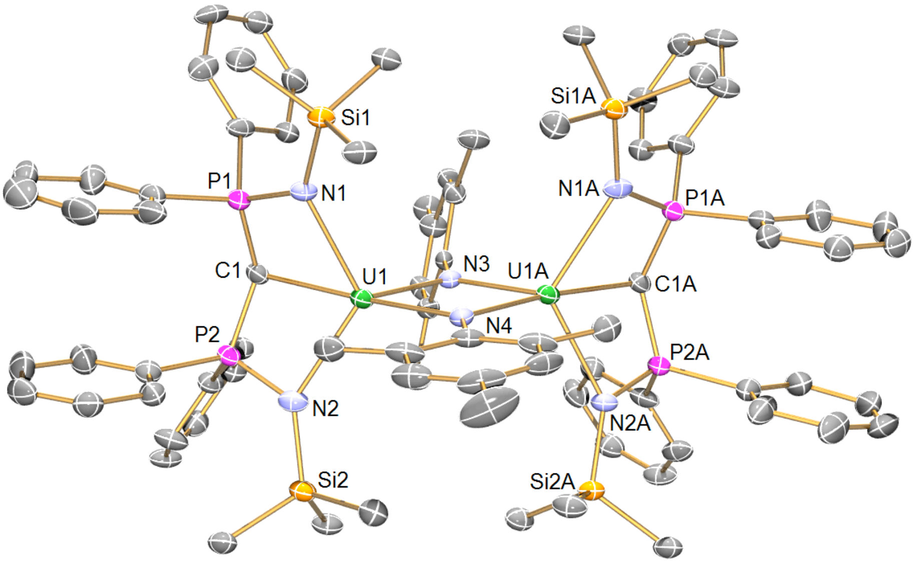

Figure S18. Molecular structure of 3 at 120 K with selective labelling and displacement ellipsoids set to 40%. Lattice solvent molecules and hydrogen atoms omitted for clarity.

Metrical parameters for the structures of compounds **2**-**6** are available free of charge from the Cambridge Crystallographic Data Centre under reference numbers CCDC 968719 (**2**), 968720 (**3**), 968721 (**4**), 968722 (**5**), and 968723 (**6**).

Calculated Frontier Orbitals of 5

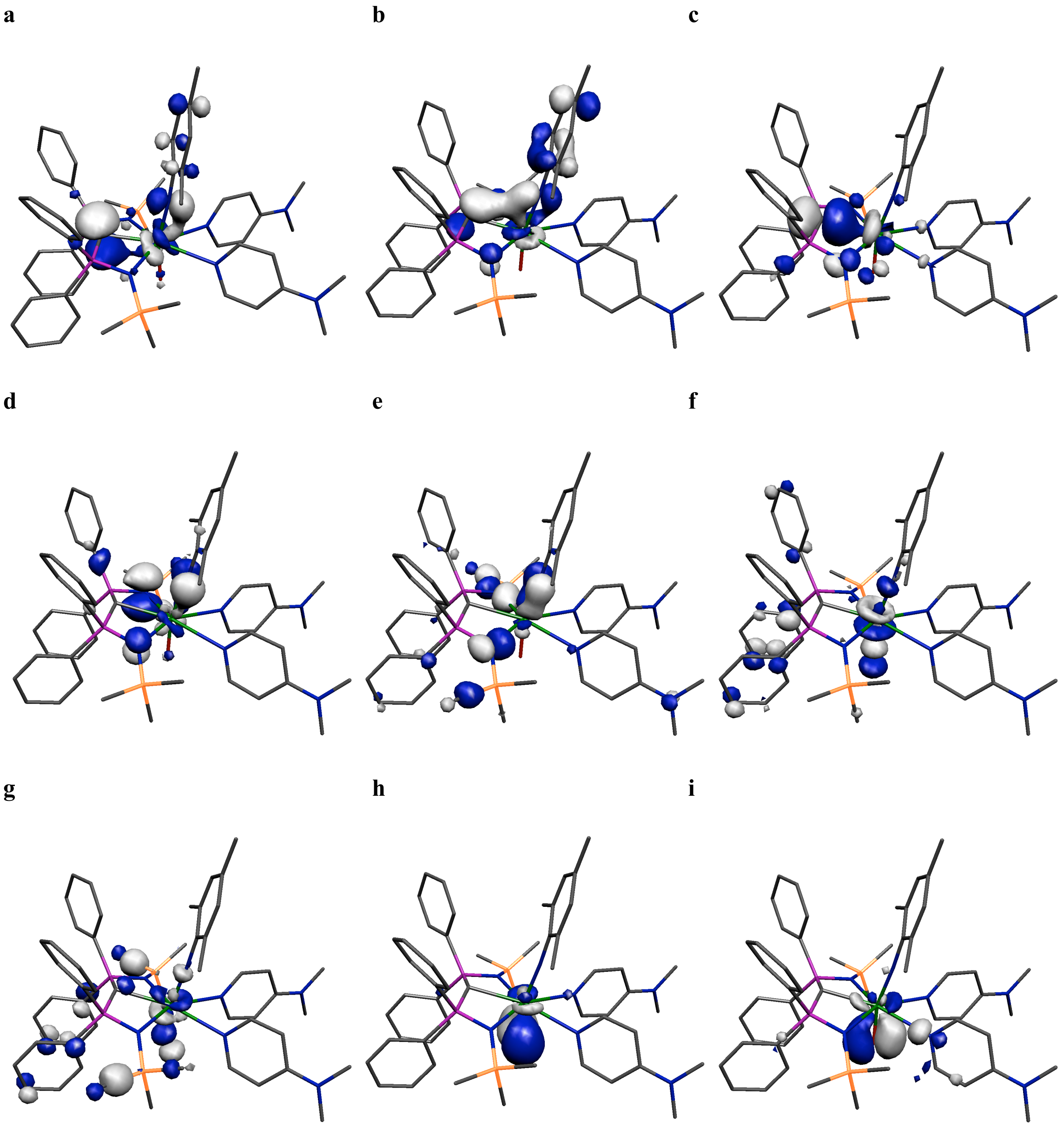

Figure S19. Selected Kohn Sham molecular orbitals of 5 with hydrogen atoms omitted. a, HOMO (300, −3.786 eV). b, HOMO−1 (299, −4.203 eV). c, HOMO −2 (298, −4.487 eV). d, HOMO−3 (297, −4.691 eV). e, HOMO−8 (292, −5.475 eV). f, HOMO−18 (282, −6.017 eV). g, HOMO−19 (281, −6.125 eV). h, HOMO−28 (272, −7.015 eV). i, HOMO−29 (271, −7.031 eV).

a b c d

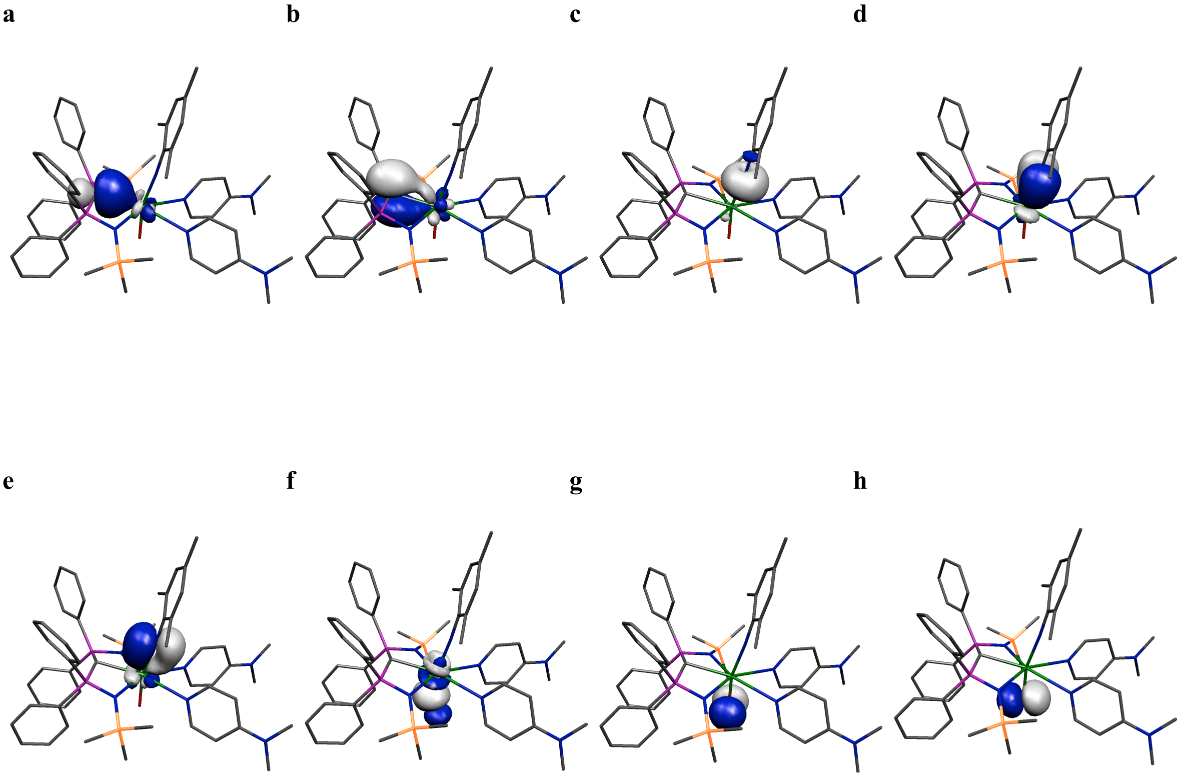

e f g h

Figure S20. Selected NBOs of 5 with hydrogen atoms omitted. a, carbene σ-bond. b, carbene π-bond. c, formal imido σ-bond. d, imido π-bond, e, imido π-bond, f, oxo σ-bond, g, formal oxo π-bond. h, formal oxo π-bond.

Calculated Frontier Orbitals of 6

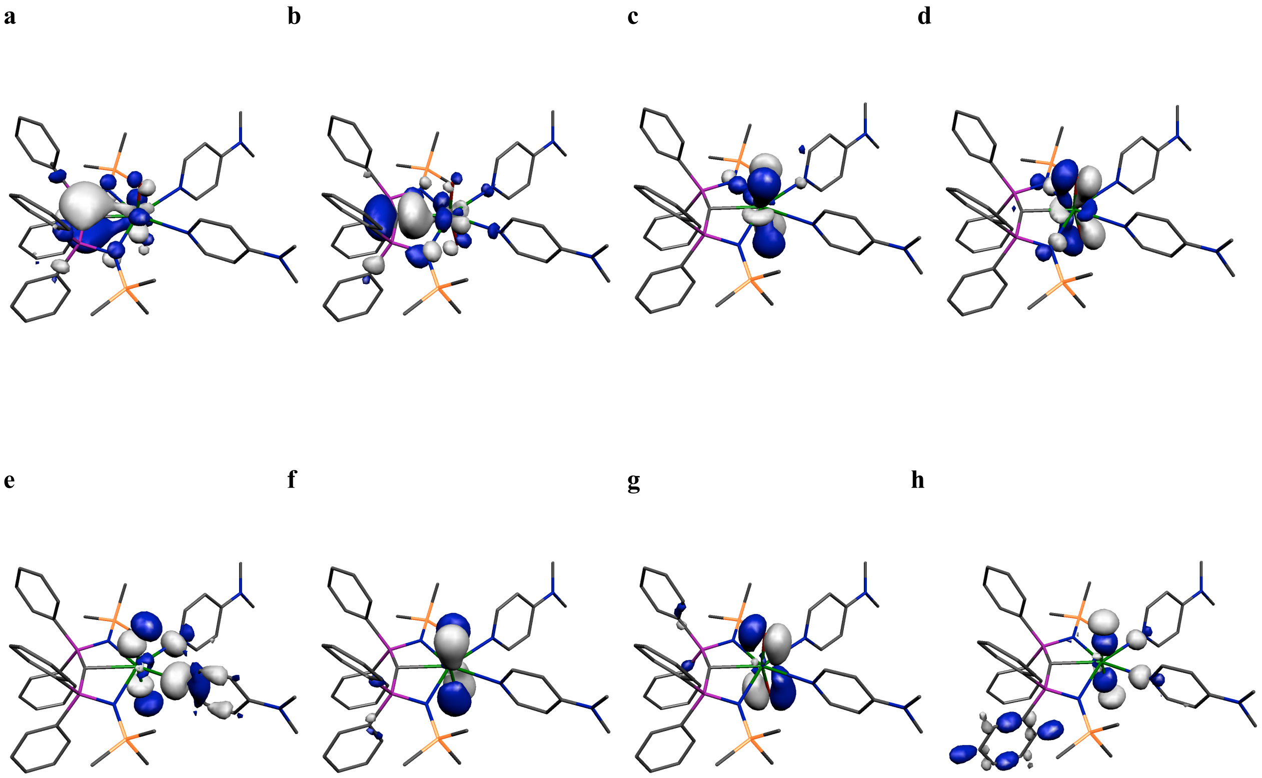

Figure S21. Selected Kohn Sham molecular orbitals of 6 with hydrogen atoms omitted. a, HOMO (268, −3.752 eV). b, HOMO−1 (267, −4.314 eV). c, HOMO−23 (245, −6.707 eV). d, HOMO−24 (244, −6.755 eV). e, HOMO−26 (242, −6.989 eV). f, HOMO−27 (241, −7.160 eV). g, HOMO−28 (240, −7.197 eV). h, HOMO−30 (238, −7.555 eV).

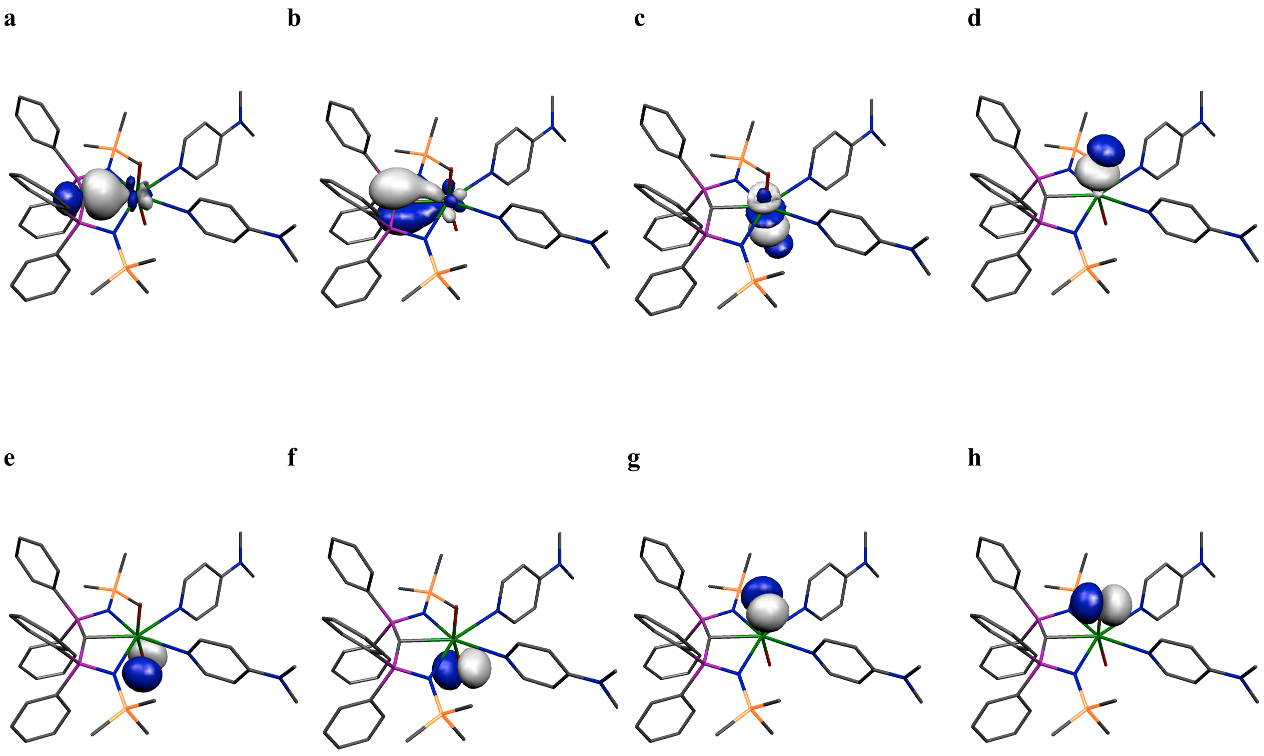

Figure S22. Selected NBOs of 6 with hydrogen atoms omitted. a, carbene σ-bond. b, carbene π-bond. c, oxo σ-bond. d, oxo σ-bond. e, formal oxo π-bond. f, formal oxo π-bond. g, formal oxo π-bond. h, formal oxo π-bond.

Table S1. Final coordinates and single point energy of 5 after geometry optimization

1.C	3.397072	-1.000204	-5.743488
2.C	4.074591	0.097787	-5.205548
3.C	2.211747	-1.445652	-5.145528
4.C	0.260683	2.781376	-4.802213
5.C	-1.625666	0.434649	-4.445713
6.C	3.571555	0.751929	-4.075620
7.C	1.712907	-0.802525	-4.012471
8.C	4.061427	-4.273887	-3.078705
9.C	2.386024	0.306951	-3.467469
10.C	-2.132337	3.119576	-2.991135
11.C	2.735615	-4.364194	-2.639869
12.C	4.908039	-3.303809	-2.531217
13.C	-7.985650	0.550642	-2.033388
14.C	1.980646	3.848241	-1.854543
15.C	2.682699	5.034531	-1.619004
16.C	-5.176197	0.151214	-1.737668
17.C	4.438731	-2.439220	-1.537933
18.C	2.264303	-3.497539	-1.651819
19.C	2.597040	2.603348	-1.673121
20.C	-3.819423	-0.057573	-1.550617
21.C	4.014219	4.986824	-1.195563
22.C	3.928089	2.563908	-1.222736

23.C	4.634650	3.747221	-0.993657
24.C	3.114998	-2.539588	-1.074814
25.C	-5.888688	0.989897	-0.843431
26.C	-7.960236	1.966452	0.037235
27.C	1.770224	0.027300	-0.595205
28.C	-5.131793	1.545752	0.219638
29.C	-3.775553	1.277871	0.312221
30.C	-1.190930	3.807744	0.522878
31.C	-0.131172	-4.754474	0.792043
32.C	-2.993722	-2.578267	0.980427
33.C	3.742761	-0.966255	1.317313
34.C	4.785572	-1.852528	1.637904
35.C	-4.041990	-3.169560	1.665946
36.C	2.599491	-4.419265	2.026776
37.C	-0.834827	3.418241	1.931901
38.C	3.678742	0.273768	1.976805
39.C	-0.470379	2.069287	2.235757
40.C	5.741384	-1.508247	2.599042
41.C	-2.677176	-1.012359	2.626420
42.C	-6.140703	-4.398959	3.165092
43.C	-4.447762	-2.642751	2.919330
44.C	-0.820423	4.379091	2.944871
45.C	4.626317	0.611834	2.947134
46.C	5.660824	-0.278170	3.261091
47.C	-3.716576	-1.521384	3.387839
48.C	0.217926	-3.273569	3.471529
49.C	-0.034909	1.760648	3.556840
50.C	0.439097	0.375884	3.918591
51.C	-0.433145	4.080937	4.260345
52.C	-5.913423	-2.574091	4.885839
53.C	-0.032507	2.769222	4.531090
54.C	-0.401804	5.154128	5.324200
55.H	3.791443	-1.510228	-6.624097
56.H	-0.394353	3.067199	-5.643170
57.H	4.999480	0.450604	-5.665249
58.H	1.677628	-2.303126	-5.558076
59.H	1.059978	2.142815	-5.207104
60.H	-0.931591	0.027227	-5.196634
61.H	-2.534893	0.765573	-4.972829
62.H	0.726339	3.701286	-4.419782
63.H	4.431284	-4.948699	-3.852815
64.H	-2.687051	3.463100	-3.879490
65.H	-1.891439	-0.388681	-3.768651
66.H	4.101868	1.616481	-3.674953
67.H	0.796674	-1.157148	-3.539518
68.H	-7.547842	0.733041	-3.025046
69.H	5.937604	-3.215457	-2.881215
70.H	2.063353	-5.106799	-3.073627
71.H	-9.010935	0.933613	-2.043896
72.H	-5.669576	-0.345212	-2.570501
73.H	-1.686551	4.006228	-2.515112
74.H	-2.858311	2.692154	-2.284696
75.H	-8.023491	-0.539676	-1.865700
76.H	0.938924	3.884963	-2.166118
77.H	-3.269268	-0.714024	-2.222280
78.H	2.185595	5.996167	-1.759855
79.H	4.563046	5.911433	-1.008096
80.H	5.107976	-1.684879	-1.122159
81.H	1.225854	-3.544402	-1.327296
82.H	4.406128	1.601813	-1.033126

83.H	5.667433	3.702302	-0.644072
84.H	-8.983474	2.145804	-0.308137
85.H	-7.497004	2.943192	0.235111
86.H	0.454364	-5.264419	0.012231
87.H	-0.437439	3.434062	-0.183031
88.H	-2.693645	-2.957117	0.004146
89.H	-2.147604	3.376629	0.196731
90.H	-8.006224	1.404672	0.986375
91.H	-0.988639	-4.272965	0.304254
92.H	-1.264669	4.899311	0.426222
93.H	-5.589846	2.176924	0.978491
94.H	4.855866	-2.817094	1.136329
95.H	3.140524	-4.618270	1.089298
96.H	-4.543568	-4.024949	1.218134
97.H	-3.193723	1.695853	1.132726
98.H	-0.516625	-5.528554	1.475265
99.H	-6.669958	-4.242014	2.210918
100.H	2.881774	0.971618	1.714799
101.H	2.375090	-5.394582	2.491156
102.H	6.548081	-2.205748	2.831566
103.H	3.268237	-3.872739	2.706868
104.H	-1.105045	5.404816	2.691790
105.H	-5.412912	-5.213847	3.026860
106.H	-2.117326	-0.147454	2.977700
107.H	0.745780	-0.188006	3.030267
108.H	-6.872480	-4.721707	3.912093
109.H	-0.800773	-2.861155	3.447516
110.H	4.557947	1.574916	3.455757
111.H	6.403907	-0.013039	4.015764
112.H	0.176126	-4.245586	3.989944
113.H	0.845256	-2.599582	4.073234
114.H	-3.942958	-1.049448	4.341629
115.H	-6.105195	-1.498038	4.758383
116.H	-0.346136	-0.211325	4.419428
117.H	1.289732	0.425929	4.611471
118.H	-6.845713	-3.044644	5.213880
119.H	0.432522	5.855519	5.163327
120.H	-1.326449	5.749893	5.324899
121.H	-5.162042	-2.699810	5.683258
122.H	0.308053	2.515113	5.539520
123.H	-0.279184	4.721132	6.325743
124.N	0.062346	1.305038	-2.100300
125.N	-7.230845	1.245631	-0.998421
126.N	-3.091831	0.498061	-0.556063
127.N	1.077504	-1.995604	0.878150
128.N	-0.556210	1.088157	1.257623
129.N	-2.289468	-1.515782	1.432374
130.N	-5.491191	-3.180422	3.630126
131.O	-1.035770	-1.554522	-1.388610
132.Si	-0.801918	1.876240	-3.516516
133.Si	0.954661	-3.516571	1.746088
134.P	1.665472	1.037888	-1.940194
135.P	2.385413	-1.361730	0.132030
136.U	-0.547006	-0.265306	-0.153059

Bond Energy: −824.69885905 eV

Table S2. Final coordinates and single point energy of 6 after geometry optimization

1.C	-3.523638	4.274583	-6.258232

2.C	-5.439620	4.141598	-4.701804
3.C	-1.856531	3.045484	-4.313886
4.C	-1.986799	-1.006762	-4.200265
5.C	-3.187347	3.418377	-3.988453
6.C	-1.075534	2.384085	-3.379054
7.C	-2.946111	-3.497822	-2.804801
8.C	-3.614051	3.085648	-2.677368
9.C	1.485154	-4.538691	-2.632009
10.C	2.880840	7.718336	-1.622895
11.C	-4.282425	-0.871567	-2.122975
12.C	0.632701	-3.564694	-2.106585
13.C	-2.747422	2.441673	-1.810246
14.C	1.791650	-5.680842	-1.883337
15.C	2.052627	4.991530	-1.465414
16.C	1.657055	3.669031	-1.348641
17.C	0.069602	-3.722767	-0.829727
18.C	1.545886	5.958738	-0.558765
19.C	1.246983	-5.839462	-0.604127
20.C	1.509008	8.205445	0.423478
21.C	0.395446	-4.864784	-0.077834
22.C	4.458268	-3.153410	0.368888
23.C	4.332910	1.600315	0.314323
24.C	0.650838	5.474546	0.430878
25.C	-2.761655	-4.376548	0.629366
26.C	3.408381	-2.253031	0.551309
27.C	0.309470	4.131679	0.452699
28.C	-2.269344	-3.076200	0.831296
29.C	0.080127	-1.343039	0.840966
30.C	4.830772	-4.020246	1.404879
31.C	-3.842072	-4.851976	1.381076
32.C	2.722906	-2.197470	1.779464
33.C	-2.887560	-2.256330	1.793667
34.C	-4.450142	-4.030109	2.335125
35.C	4.149430	-3.976609	2.624758
36.C	-3.971948	-2.728718	2.536377
37.C	2.962915	3.018892	2.674115
38.C	3.102985	-3.067082	2.814103
39.C	4.391678	0.411182	3.121486
40.C	0.770199	-1.077492	3.649663
41.C	0.064504	-2.201393	4.113436
42.C	0.948746	0.012482	4.512352
43.C	-0.428795	-2.241927	5.420003
44.C	0.453582	-0.023431	5.820578
45.C	-0.233345	-1.152393	6.278869
46.H	-4.249663	4.895569	-6.793868
47.H	-3.406693	3.323504	-6.807097
48.H	-2.561436	4.802920	-6.263454
49.H	-5.947510	3.226149	-5.054752
50.H	-1.437294	3.234767	-5.299208
51.H	-5.835981	4.999745	-5.258922
52.H	-2.698753	-1.294470	-4.991306
53.H	-1.008393	-1.441887	-4.455968
54.H	-1.885225	0.087028	-4.223469
55.H	-5.689236	4.290507	-3.647772
56.H	-3.603218	-3.593792	-3.685752
57.H	-0.066136	2.064302	-3.637649
58.H	1.914761	-4.404404	-3.626191
59.H	-2.033459	-4.079418	-3.004156
60.H	2.557217	7.451528	-2.639594
61.H	-5.027716	-1.146976	-2.887258

62.H	0.405269	-2.662103	-2.673339
63.H	-4.616108	3.313704	-2.321954
64.H	2.762024	5.258649	-2.245719
65.H	-4.236039	0.225835	-2.078017
66.H	-3.468281	-3.959163	-1.954133
67.H	2.973793	8.807837	-1.576098
68.H	2.461352	-6.439967	-2.290830
69.H	2.047912	2.913896	-2.030578
70.H	3.876685	7.277410	-1.447528
71.H	-4.645355	-1.236767	-1.149805
72.H	-3.061188	2.190765	-0.797534
73.H	4.982655	-3.185175	-0.587327
74.H	3.794515	2.195582	-0.437097
75.H	1.835252	9.215333	0.155674
76.H	-2.301610	-5.024084	-0.117000
77.H	4.596163	0.637112	-0.147997
78.H	3.110760	-1.585071	-0.258141
79.H	1.495155	-6.718926	-0.007905
80.H	0.416637	8.227257	0.545191
81.H	5.269590	2.126733	0.560014
82.H	5.648216	-4.728927	1.259191
83.H	1.962538	7.938635	1.393118
84.H	-0.008532	-4.990776	0.927521
85.H	-4.208909	-5.866661	1.217102
86.H	0.224506	6.133624	1.184355
87.H	-0.382740	3.747603	1.201474
88.H	-2.501115	-1.249336	1.957849
89.H	2.401082	3.699398	2.018448
90.H	4.786567	-0.536146	2.726180
91.H	3.924265	3.502704	2.914612
92.H	-5.292846	-4.401948	2.921270
93.H	4.434707	-4.647253	3.437311
94.H	5.251435	1.058217	3.365386
95.H	-4.439506	-2.082178	3.281113
96.H	-0.119242	-3.040154	3.440162
97.H	2.400515	2.921541	3.616145
98.H	2.590738	-3.028412	3.776308
99.H	3.870211	0.187501	4.064865
100.H	1.459235	0.901043	4.145776
101.H	-0.980481	-3.118856	5.762826
102.H	0.597712	0.834981	6.479292
103.H	-0.626524	-1.180290	7.296966
104.N	-3.994239	4.078183	-4.891540
105.N	-1.489211	2.065943	-2.134180
106.N	-1.420132	-1.302438	-1.276439
107.N	1.899531	7.283856	-0.636899
108.N	0.794871	3.213870	-0.413079
109.N	1.792347	0.512576	1.475817
110.O	1.191767	0.471267	-1.718323
111.O	-1.333604	1.376867	0.751718
112.Si	-2.601263	-1.651224	-2.525214
113.Si	3.292047	1.327319	1.873869
114.P	-0.882875	-2.320724	-0.121906
115.P	1.337066	-0.993176	1.897096
116.U	-0.045345	0.703095	-0.374214

Bond Energy: −704.89324093 eV

General Computational Details

Geometry optimizations were performed as appropriate for full models of **5** and **6** using coordinates derived from their X-ray crystal structures. No constraints were imposed on the structures during the geometry optimizations. The calculations were performed using the Amsterdam Density Functional (ADF) suite version 2012.01.[4,5] The DFT geometry optimizations employed Slater type orbital (STO) triple-ζ-plus polarization all-electron basis sets (from the ZORA/TZP database of the ADF suite). Scalar relativistic approaches were used within the ZORA Hamiltonian for the inclusion of relativistic effects and the local density approximation (LDA) with the correlation potential due to Vosko et al[6] was used in all of the calculations. Gradient corrections were performed using the functionals of Becke[7] and Perdew.[8] MOLEKEL[9] was used to prepare the three-dimensional plot of the electron density. Natural Bond Order (NBO) analyses were carried out with NBO 5.0.[10] The Atoms in Molecules analysis was carried out with Xaim-1.0.[11-13] The UV/Vis/NIR electronic absorption spectrum of **5** was calculated using TD-DFT with the SAOP functional on the geometry optimized coordinates from the calculations above. The calculated structures compare very well to the experimental structures with bond lengths and angles computed to within 0.05 Å and 2°. Calculated charges for **6**: U = +3.40; $C_{carbene}$ = −1.96; O = −0.81 and −0.87. Nalewajski-Mrozek bond indices: U=C = 1.23; U=O = 2.65 and 2.66. Calculated bond lengths: U=C = 2.3831 Å; U=O = 1.8414 and 1.8388 Å.

References

1. O. J. Cooper, D. P. Mills, J. McMaster, F. Moro, E. S. Davies, W. Lewis, A. J. Blake, S. T. Liddle, *Angew. Chem. Int. Ed.* **2011**, *50*, 2383.
2. P. J. Bailey, R. A. Coxall, C. M. Dick, S. Fabre, L. C. Henderson, C. Herber, S. T. Liddle, D. Loroño-González, A. Parkin and S. Parsons, *Chem. Eur. J.* **2003**, *9*, 4820.
3. C. A. Laskowski, G. L. Hillhouse, *Organometallics* **2009**, *28*, 6114.
4. C. Fonseca Guerra, J. G. Snijders, G. te Velde E. J. Baerends, *Theor. Chem. Acc.* **1998**, *99*, 391.
5. G. te Velde, F. M. Bickelhaupt, S. J. A. van Gisbergen, C. Fonseca Guerra, E. J. Baerends, J. G. Snijders T. Ziegler, *J. Comput. Chem.* **2001**, *22*, 931.
6. S. H. Vosko, L. Wilk, M. Nusair, *Can. J. Phys.* **1980**, *58*, 1200.
7. A. D. Becke, *Phys. Rev. A.* **1988**, *38*, 3098.
8. J. P. Perdew, *Phys. Rev. B.* **1986**, *33*, 8822.
9. S. Portmann, H. P. Luthi, *Chimia* **2000**, *54*, 766.
10. NBO 5.0: E. D. Glendening, J. K. Badenhoop, A. E. Reed, J. E. Carpenter, J. A. Bohmann, C. M. Morales, F. Weinhold, (Theoretical Chemistry Institute, University of Wisconsin, Madison, WI, 2001); http://www.chem.wisc.edu/~nbo5.
11. R. F. W. Bader, *Atoms in Molecules: A Quantum Theory*, Oxford University Press, New York, 1990.
12. R. F. W. Bader, *J. Phys. Chem. A.* **1998**, *102*, 7314.
13. http://www.quimica.urv.es/XAIM.
